# Recovery of Depleted miR-146a in ALS Cortical Astrocytes Reverts Cell Aberrancies and Prevents Paracrine Pathogenicity on Microglia and Motor Neurons

**DOI:** 10.3389/fcell.2021.634355

**Published:** 2021-04-23

**Authors:** Marta Barbosa, Cátia Gomes, Catarina Sequeira, Joana Gonçalves-Ribeiro, Carolina Campos Pina, Luís A. Carvalho, Rui Moreira, Sandra H. Vaz, Ana Rita Vaz, Dora Brites

**Affiliations:** ^1^Instituto de Investigação do Medicamento (iMed.ULisboa), Faculdade de Farmácia, Universidade de Lisboa, Lisbon, Portugal; ^2^Instituto de Medicina Molecular João Lobo Antunes, Faculdade de Medicina, Universidade de Lisboa, Lisbon, Portugal; ^3^Instituto de Farmacologia e Neurociências, Faculdade de Medicina, Universidade de Lisboa, Lisbon, Portugal; ^4^Departamento de Ciências Farmacêuticas e do Medicamento, Faculdade de Farmácia, Universidade de Lisboa, Lisbon, Portugal

**Keywords:** amyotrophic lateral sclerosis, astrocyte-microglia communication, astrocyte-motor neuron crosstalk, calcium signaling aberrancies, glycoursodeoxycholic acid, reactive astrocytes, small extracellular vesicles, vinyl sulfone

## Abstract

Reactive astrocytes in Amyotrophic Lateral Sclerosis (ALS) change their molecular expression pattern and release toxic factors that contribute to neurodegeneration and microglial activation. We and others identified a dysregulated inflammatory miRNA profile in ALS patients and in mice models suggesting that they represent potential targets for therapeutic intervention. Such cellular miRNAs are known to be released into the secretome and to be carried by small extracellular vesicles (sEVs), which may be harmful to recipient cells. Thus, ALS astrocyte secretome may disrupt cell homeostasis and impact on ALS pathogenesis. Previously, we identified a specific aberrant signature in the cortical brain of symptomatic SOD1-G93A (mSOD1) mice, as well as in astrocytes isolated from the same region of 7-day-old mSOD1 mice, with upregulated S100B/HMGB1/Cx43/vimentin and downregulated GFAP. The presence of downregulated miR-146a on both cases suggests that it can be a promising target for modulation in ALS. Here, we upregulated miR-146a with pre-miR-146a, and tested glycoursodeoxycholic acid (GUDCA) and dipeptidyl vinyl sulfone (VS) for their immunoregulatory properties. VS was more effective in restoring astrocytic miR-146a, GFAP, S100B, HMGB1, Cx43, and vimentin levels than GUDCA, which only recovered Cx43 and vimentin mRNA. The miR-146a inhibitor generated typical ALS aberrancies in wild type astrocytes that were abolished by VS. Similarly, pre-miR-146a transfection into the mSOD1 astrocytes abrogated aberrant markers and intracellular Ca^2+^ overload. Such treatment counteracted miR-146a depletion in sEVs and led to secretome-mediated miR-146a enhancement in NSC-34-motor neurons (MNs) and N9-microglia. Secretome from mSOD1 astrocytes increased early/late apoptosis and FGFR3 mRNA in MNs and microglia, but not when derived from pre-miR-146a or VS-treated cells. These last strategies prevented the impairment of axonal transport and synaptic dynamics by the pathological secretome, while also averted microglia activation through either secretome, or their isolated sEVs. Proteomic analysis of the target cells indicated that pre-miR-146a regulates mitochondria and inflammation via paracrine signaling. We demonstrate that replenishment of miR-146a in mSOD1 cortical astrocytes with pre-miR-146a or by VS abrogates their phenotypic aberrancies and paracrine deleterious consequences to MNs and microglia. These results propose miR-146a as a new causal and emerging therapeutic target for astrocyte pathogenic processes in ALS.

## Introduction

Amyotrophic Lateral Sclerosis (ALS) is a neurodegenerative disease with a life expectancy of only 3 years. It is unclear where the disease originates, or which are the specific targets, mediators, and the underlying ALS mechanisms involved, hindering the search for an effective therapeutic strategy. Though the majority of cases occurs in people with no prior family history, several mutations in different genes were already identified (Mejzini et al., 2019), and the mutant Cu/Zn superoxide dismutase 1 (mSOD1) mouse is the mostly widely used animal model in research. This model is well characterized in terms of behavior, histopathology, and molecular mechanisms, and recapitulates most of the pathological features of the human ALS (Al-Chalabi et al., 2017). Although motor neuron (MN) loss is believed to be the predominant feature, astrogliosis has a key role in MN degeneration due to the release of unknown toxic factors, either in the brain cortex or in the spinal cord (SC) (Robberecht and Philips, 2013; Gomes et al., 2020), and in both sporadic ALS (sALS) (Varga et al., 2018) and familiar ALS (fALS) (Meyer et al., 2014; van Rheenen et al., 2016).

Astrocytes isolated from the SC of symptomatic rats exhibit low levels of glial fibrillary acidic protein (GFAP) and glutamate transporter 1 (GLT-1), together with increased S100B and Cx43, and were designated as aberrant astrocytes (Díaz-Amarilla et al., 2011). We lately confirmed this same set of aberrant/reactive markers in astrocytes isolated from the SOD1G93A (mSOD1) mice at 7-day-old and cultured for 13 days *in vitro* (DIV) (Gomes et al., 2019; Gomes et al., 2020), pointing that such astrocytes acquire early deficits associated to a disease-specific phenotype, including low levels of GFAP. These low levels of GFAP were also found in other harmful conditions (Escartin et al., 2021).

The secretome from ALS aberrant astrocytes was shown to potentiate MN death (Díaz-Amarilla et al., 2011; Trias et al., 2017; Gomes et al., 2019), and to lead to activated microglia (Filipi et al., 2020), but the underlying neurotoxic processes and how the factors involved are disseminated are still to be clarified. One of the mechanisms may relate with the secretion of small extracellular vesicles (sEVs) from mSOD1 astrocytes with miRNAs (Jovicic and Gitler, 2017) and mSOD1 protein (Basso et al., 2013) that showed to cause the selective death of MNs. We previously found that the aberrant mSOD1 cortical astrocytes expressed reduced levels of miR-146a (Gomes et al., 2019). We also identified reduced levels of miR-146a in sEVs released from both cortical and spinal 13 DIV astrocytes that when cocultured with MNs showed to determine several dysfunctionalities (Gomes et al., 2020). We then wonder whether such depleted levels of miR-146a in mSOD1 astrocytes and their sEVs could relate with the cell pathological features and have harmful consequences to nearby MNs and microglia. In conformity, recent data with astrocytes directly converted from the C9orf72 patient fibroblasts has shown that their released sEVs with downregulated miR-494-3p caused neuronal network degeneration (Varcianna et al., 2019).

miRNAs are a class of small non-coding RNAs that play important roles in regulating gene expression. Among them some are more closely associated with inflammatory pathways, i.e., miR-155, miR-146a, miR-21 and miR-124, and are designed as inflamma-miRNAs (Brites, 2020). Their dysregulation was found in ALS (Butovsky et al., 2012; De Felice et al., 2014; Freischmidt et al., 2014), and they were suggested to be potential biomarkers (Ricci et al., 2018; Joilin et al., 2019). Upregulation of miR-155 was found in fALS and sALS patients (Koval et al., 2013; Butovsky et al., 2015), as well as in the SC of mSOD1 mice, in either pre-symptomatic or symptomatic stages (Cunha et al., 2018). When targeted in microglia, its reduction counteracted microglia activation and attenuated the disease in the mSOD1 mice (Butovsky et al., 2015). Our discovered depletion of miR-146a in aberrant cortical astrocytes and their sEVs may also determine a polarized pro-inflammatory microglia phenotype if we consider that it has a key role in repressing inflammation (Taganov et al., 2006; Iyer et al., 2012; Waters et al., 2019).

In this study we decided to use different strategies to recover the normal levels of miR-146a in the ALS aberrant cortical astrocytes, comprising either the pre-miR-146a or compounds recognized as immunoregulators. Our previous data evidenced that glycoursodeoxycholic acid (GUDCA) and dipeptidyl vinyl sulfone (VS) are effective in regulating inflammatory miRNAs and counteracting the release of pro-inflammatory cytokines (Fernandes et al., 2007; Vaz et al., 2015, 2019; Falcão et al., 2017). Thus, we hypothesized that replenishment of miR-146a in the cortical mSOD1 astrocytes toward normal levels could abrogate their phenotypic aberrancies. If that happens, we will avoid the release of neurotoxic factors leading to MN distress and microglia activation (Nagai et al., 2007; Ajmone-Cat et al., 2019; Gomes et al., 2019). Such polarized microglia release TNF-α what in turn further contributes to astrocyte reactivity and neurotoxicity (Liddelow and Barres, 2017). Therefore, restoration of the mSOD1 astrocyte steady-state phenotype will facilitate homeostatic balance recovery. This is important, once astrocyte-microglia crosstalk regulation or deregulation determines neuronal functions and dysfunctions (Jha et al., 2018). Actually, elimination of IL-1α, TNF-α, and C1q in the mSOD1 mice, extended their survival (Guttenplan et al., 2020), highlighting astrocyte reactivity and microglia activation as therapeutic targets to prevent neurodegeneration in ALS.

In this work we used the cortical astrocytes isolated from 7-day-old mSOD1 mice and cultured for 13 DIV, that were transfected with pre-miR-146a or treated with the immunomodulators GUDCA and VS, to test their ability to abolish miR-146a downregulation. Astrocyte reactivity markers usually characterizing their aberrant phenotype, together with the Ca^2+^ overload (Kawamata et al., 2014), were assessed with or without treatments. In addition, we evaluated the potential neuroprotective and anti-inflammatory properties of the secretome from treated astrocytes *vs.* the untreated ones, using the mouse NSC-34 MN-like cell line produced from the SC and the N9 microglial cell line from the brain. We explored the expression of synaptic and axonal genes in MNs, iNOS and TNF-α genes in microglia, as well as cell death by apoptosis, fibroblast growth factor receptors (FGFR) 1 and 3 mRNAs, miR-146a and proteomics in both cell types.

Our study is the first targeting the depressed levels of miR-146a in cortical ALS astrocytes toward its restoration, with ensuing data highlighting that both pre-miR-146a and VS may represent new therapeutic ALS strategies to reestablish astrocyte neuroprotection and microglia/MN homeostatic balance. Revival of disease-specific cortical astrocytes in ALS was found to be associated to the replenishment of miR-146a.

## Materials and Methods

### Ethics Statement

The present study was performed in accordance with the European Community guidelines (Directives 86/609/EU and 2010/63/EU, Recommendation 2007/526/CE, European Convention for the Protection of Vertebrate Animals used for Experimental or Other Scientific Purposes ETS 123/Appendix A) and Portuguese Laws on Animal Care (Decreto-Lei 129/92, Portaria 1005/92, Portaria 466/95, Decreto-Lei 197/96, Portaria 1131/97). All the protocols used in this study were approved by the Portuguese National Authority (General Direction of Veterinary) and the Ethics Committee of the Instituto de Medicina Molecular João Lobo Antunes (IMM) from Faculty of Medicine, University of Lisbon, Portugal. Every effort was made to minimize the number of animals used and their suffering, according with the 3R’s principle.

### Transgenic SOD1-G93A Mouse Model

Transgenic B6SJL-TgN (SOD1-G93A)1Gur/J males (Jackson Laboratory, no. 002726) overexpressing the human(h)SOD1 gene carrying a glycine to alanine point mutation at residue 93 (mSOD1) (Gurney, 1994) and B6SJLF1/J non-transgenic wild type (WT) females were purchased from The Jackson Laboratory (Bar Harbor, ME, United States). Maintenance and handling took place at IMM animal house facilities, where a colony was established. Mice were maintained on a background B6SJL by breeding SOD1-G93A transgenic males with non-transgenic females. Both males and females were used as described by us (Gomes et al., 2020). Transgenic SOD1-G93A mice were compared to aged-matched WT mice. All animals were maintained on 12 h light/12 h dark cycle and received food and water *ad libitum*. Average number of animals per cage was 4 to 5.

### Primary Culture of Astrocytes

Astrocytes were isolated from the cerebral cortices of WT and mSOD1 mice at postnatal day 7 (Gomes et al., 2019, 2020). Approximately, 2.0 × 10^5^ cells/cm^2^ were plated on tissue culture plates in culture medium [DMEM (Biochrom AG, Berlin, Germany) supplemented with 11 mM sodium bicarbonate (Merck, Darmstadt, Germany), 38.9 mM glucose, 1% Antibiotic-Antimycotic solution (Sigma-Aldrich, St. Louis, MO, United States) and 10% Fetal Bovine Serum (FBS) (Biochrom AG)] and maintained at 37°C in a humidified atmosphere of 5% CO2 until 13 DIV, with changing of the culture medium at 7 DIV and 10 DIV. Then, cells were used for the evaluation of reactive and inflammatory mediators, with or without treatment strategies. These cells previously showed a set of specific-disease markers that were also found in symptomatic mice (Gomes et al., 2019), highlighting an early presentation of the mSOD1-associated disease phenotype in such neonatal astrocytes. Moreover, the culturing for 13 DIV guarantees the maturation of astrocytes, as we previously showed (Falcão et al., 2005, 2006). To assess astrocyte paracrine signaling, secretomes were collected from 13 DIV astrocytes, either modulated or not (as next explained), and sEVs were isolated.

### Modulation of Cortical Astrocyte Aberrancies

At 12 DIV, cells were incubated with 50 μM GUDCA (Calbiochem; Darmstadt, Germany) or 10 μM VS (synthetized by the Medicinal Chemistry group from Instituto de Investigação do Medicamento) (Falcão et al., 2017), and maintained for further 24 h.

To downregulate miR-146a in WT astrocytes, we transfected cells with 15 nM Anti-miR^TM^ 146a inhibitor (#AM10722, Ambion^®^, Thermo Fisher Scientific, Waltham, MA, United States) at 11 DIV. According with manufacturer’s description, they are chemically modified single-stranded oligonucleotides with a patented secondary structure that binds and inhibits the endogenous miR-146a.

To upregulate miR-146a in depleted mSOD1 astrocytes, the cells were transfected with 15 nM Pre-miR^TM^ 146a Precursor (#PM10722, Ambion^®^), a chemically modified double-stranded RNA designed to mimic the endogenous precursor miR-146a and to ensure the uptake of the correct strand to the RISC complex. The mature miR-146a sequence was 5′UGAGAACUGAAUUC CAUGGGUU3′ (hsa-miR-146a-5p). Then, the oligonucleotides were mixed with X-tremeGENE^TM^ HP DNA Transfection Reagent (Sigma-Aldrich, St. Louis, MO, United States) in a proportion 2:1 and diluted in Opti-MEM^TM^ (Thermo Scientific, Waltham, MA, United States). The controls used were the non-transfected WT and mSOD1 cells treated only with X-tremeGENE^TM^ HP DNA Transfection Reagent diluted in Opti-MEM^TM^ (mock control). To make sure that the effect of miRNA-146a transfection in cells was due to its specificity, we also transfected WT cells with 15 nM of Anti-miR Negative Control and mSOD1 ones with 15 nM Pre-miR Negative Control (Ambion^®^). Results from mock controls, and respective Anti-miR Negative Control and Pre-miR Negative Control, were similar, validating the miR-146a specificity (Supplementary Table 1). After 12 h, medium was changed [DMEM supplemented with 11 mM sodium bicarbonate, 38.9 mM glucose, 1% FBS sEVs-depleted and 1% Antibiotic-Antimycotic solution] and maintained for 24 h. To evaluate which alterations in astrocyte phenotype treated with VS involved miR-146a regulation, we transfected WT astrocytes with 15 nM anti-miR-146a inhibitor at 11 DIV. After 12 h the medium was changed, and the VS compound was added. The cells were maintained in culture for further 24 h.

### Culture of NSC-34 MN-Like Cells and Treatment With Astrocyte-Derived Secretome

In the present study we used the neuroblastoma hybrid cell line NSC-34 expressing the human SOD1 WT. These cells, produced by the fusion of MNs from mouse embryos with mouse neuroblastoma cells N18TG2 (Cashman et al., 1992), were shown to reproduce many features of MNs when differentiated and maturated, and have been used to assess neurotoxicity/neuroprotection (Maier et al., 2013; Vaz et al., 2015), including astrocyte-induced neurodegeneration (Benkler et al., 2013). As before, we decided to use these cells given our previous experience with them (Vaz et al., 2015; Pinto et al., 2017; Gomes et al., 2019) and considering the limitations of primary spinal MN cultures, and the immature phenotype of those generated from embryonic stem cells or from induced pluripotent stem cells (Ho et al., 2016; Bucchia et al., 2018).

NSC-34 cells were cultured in Poly-D-lysine coated culture plates at a density of 5 × 10^4^ cells/ml and maintained for 48 h in the proliferation media [DMEM high glucose, w/o pyruvate, supplemented with 10% of FBS and 1% of penicillin/streptomycin, and geneticin sulfate (G418, 0.5 mg/ml) for selection], and then for additional 4 DIV in the differentiation media [DMEM-F12 plus FBS (1%), non-essential amino acids (1%) and penicillin/streptomycin (1%)], as previously described (Vaz et al., 2015). Media from the differentiated NSC-34 cells was removed and the cells incubated for 48 h with an equivalent volume of the secretome derived from untreated astrocytes and from pre-miR-146a and VS treated cells.

### N9-Microglia Cell Culture and Treatment With Astrocyte-Derived Secretome and sEVs

Mouse microglial N9 cell line results from the immortalization of microglia isolated from the cortex of CD1 mouse embryos (Righi et al., 1989). This cell line shows properties that are analogous to primary cultures of microglia, such as migration, phagocytosis, and inflammation-related features (Fleisher-Berkovich et al., 2010), and respond similarly to lipopolysaccharide (LPS)-induced activation (Nikodemova and Watters, 2011; Cunha et al., 2016).

N9 microglial cells were plated on uncoated culture plates at a density of 1 × 10^5^ cells/ml and maintained for 24 h in the culture medium [RPMI media (Sigma-Aldrich, St. Louis, MO, United States) supplemented with 10% FBS, 1% L-glutamine, and 1% antibiotic penicillin/streptomycin (1%), all from Biochrom AG, Berlin, Germany], as usual in our laboratory (Cunha et al., 2016; Pinto et al., 2017; Vaz et al., 2019). Then, culture media were removed, and the cells incubated for 24 h with an equivalent volume of the secretome derived from untreated astrocytes and from pre-miR-146a and VS treated cells. Secretome contains soluble factors and vesicular fractions as sEVs, essentially containing miRNAs (Pinho et al., 2020). We showed that sEVs were preferentially collected by microglia than by MNs, when in coculture (Pinto et al., 2017). To assess whether the treatment of astrocytes with pre-miR-146a or with VS would influence the number of sEVs engulfed by the N9 microglia, we incubated the isolated sEVs with microglia for 24 h. Subsequently, we assessed the influence of those sEVs in shifting the inflammatory state of microglia.

### sEV Isolation, Characterization, and Labeling

sEVs were isolated from the secretome of WT and mSOD1 astrocytes, using the process implemented in our lab (Pinto et al., 2017). Briefly, the culture media was centrifuged at 1,000 g for 10 min to remove cell debris. After that, the supernatant was centrifuged at 16,000 g to separate large extracellular vesicles (lEVs, size 1,000 nm) and, the recovered supernatant was passed into a 0.22 μM filter and further centrifuged in the Ultra L-XP100 centrifuge (Beckman Coulter Inc., CA, United States) at 100,000 g to pellet sEVs, as we previously detailed (Pinto et al., 2017). Characterization of sEVs in terms of shape was performed using Negative-Staining Transmission Electron Microscopy (Jeol Jem 1400 TEM 120 kV, Tokyo, Japan). Concentration and size of sEVs were evaluated by Nanoparticle tracking analysis (NTA) using the Nanosight (model LM10-HSBF, Malvern, United Kingdom). Expression of alix and flotillin-1 was assessed by Western blot analysis, using 30 μg of total protein (antibodies are indicated in the Supplementary Table 2). To evaluate miRNA cargo, the final pellet containing sEVs was resuspended in lysis buffer, and the RNA extracted as explained below.

To monitor the incorporation of astrocyte-derived sEVs by N9 microglia, the sEVs were labeled with the PKH67 Fluorescent Linker Kit (Sigma Aldrich) in accordance with manufacturer specifications, resuspended in DMEM with 1% penicillin/streptomycin and added to N9 microglia cultures.

### Immunocytochemistry

At 13 DIV, astrocytes were fixed with 4% paraformaldehyde and immunofluorescence staining performed as published (Gomes et al., 2019). Briefly, cells were incubated overnight at 4°C with GFAP (listed in the Supplementary Table 2). In the following day, cells were incubated with secondary antibody, also referred in the Supplementary Table 2. Cell nuclei were stained with Hoechst 33258 dye and then mounted onto uncoated slides using PBS-Glycerol (1:1). Fluorescence was visualized using an AxioCam HR camera adapted to an AxioScopeA1 microscope.

Fluorescence of ten random microscopic fields were acquired per sample using Zen (blue edition, 2012, Zeiss) software and the number of positive cells above a determined threshold or fluorescence per total number of cells was quantified. Area of sEVs labeled with PKH67 was determined per total number of PKH67-positive cells.

### Western Blot Analysis

Cell lysis and protein isolation was made as usual in our lab (Gomes et al., 2019), and we used the protein assay kit (Bio-Rad, Hercules, CA, United States) to assess the protein concentration as manufacturer’s specifications. Then, equal amounts of protein were subjected to SDS-PAGE and transferred to a nitrocellulose membrane. After blocking with 5% (w/v) non-fat milk solution, nitrocellulose membranes were incubated overnight at 4°C with primary antibodies (depicted in the Supplementary Table 2). In the following day, secondary antibodies conjugated to horseradish peroxidase were used (Supplementary Table 2). The chemiluminescent detection was performed after membrane incubation with LumiGLO^®^ (Cell Signaling). The relative intensities of protein bands were analyzed using the Image Lab^TM^ analysis software, after scanning with ChemiDocXRS, both from Bio-Rad Laboratories (Hercules, CA, United States). Results in cells were normalized to β-actin expression levels.

### RT-qPCR

Cellular RNA was isolated using TripleXtractor Reagent (GRISP, Porto, Portugal) (Gomes et al., 2019). For astrocyte-derived sEVs, RNA was extracted using miRNeasy Mini kit (Qiagen, Hilden, Germany). Quantification was performed using Nanodrop ND-100 Spectrophotometer (NanoDrop Technologies, Wilmington, DE, United States).

For gene expression, cellular RNA was converted to complementary DNA (cDNA) using GRS cDNA Synthesis Mastermix Kit (GRISP). Reverse transcriptase quantitative PCR (RT-qPCR) was accomplished using Xpert Fast SYBR Green Blue (GRISP) and the following optimized conditions: 94°C for 2 min followed by 40 cycles at 95°C for 0.05 min and 62°C for 0.3 min. To verify the specificity of the amplification, a melt-curve analysis was performed immediately after the amplification protocol. Non-specific products of PCR were not found in any case. β-actin was used as an endogenous control to normalize the expression levels. Primer sequences are listed in the Supplementary Table 3.

Expression of miRNA-146a (5′ UGAGAACUGAAUUCCAU GGGUU 3′) was assessed in cells and sEVs by RT-qPCR. After RNA quantification, cDNA conversion for miRNA quantification was performed with the miRCURY LNA^TM^ RT Kit (Qiagen), using 5 ng total RNA for cells and 10 ng for sEVs. The PowerUp^TM^ SYBR^TM^ Green Master Mix (Applied Biosystems, Life Technologies) was used in combination with pre-designed primers. The reaction conditions consisted of polymerase activation/denaturation and well-factor determination at 95°C for 10 min, followed by 50 amplification cycles at 95°C for 10 s and 60°C for 1 min (ramp-rate of 1.6°/s). SNORD110 (reference gene) was used as an endogenous control and to normalize the expression levels in cells. Both SNORD110 and spike-in were used as endogenous controls and geometric mean from both were calculated to normalize the expression levels in sEVs. RT-qPCR was accomplished on a QuantStudio 7 Flex Real-Time PCR System (Applied Biosystems, Life Technologies) and 384-well plates were used. Relative mRNA/miRNA concentrations were calculated using the 2^–ΔΔ*CT*^ method.

### Cell Death Determination

Phycoerythrin-conjugated Annexin V (Annexin V-PE) and 7-amino-actinomycin D (7-AAD; Guava Nexin^®^ Reagent, no. 4500-0450, Millipore) were used to determine the percentage of viable (Annexin V-PE and 7-AAD negative), early apoptotic (Annexin V-PE positive and 7-AAD negative), and late apoptotic/necrotic (Annexin V-PE and 7-AAD positive) cells by flow cytometry, as described (Gomes et al., 2020). After incubation, cells were trypsinized and added to the cells already detached in the culture medium. After centrifugation, the pellet of cells was resuspended in PBS containing 1% bovine serum albumin. Then, they were stained with Guava Nexin Reagent according to manufacturer’s instructions and analyzed on a Guava easyCyte 5HT flow cytometer (Guava Nexin^®^ Software module, Millipore).

### Ca^2+^ Imaging in Astrocytes and Analysis

Astrocyte Ca^2+^ imaging was performed as previously reported (Morais et al., 2017). Briefly, the suspension of astrocytes was plated on μ-slide 8 well chamber slide (Ibidi^®^, Gräfelfing, Germany) coated with 10 μg/ml of poly-D-lysine. At 13 DIV, cells were incubated at 37°C for 45 min with the Ca^2+^ sensitive fluorescent dye fura-2 acetoxymethyl ester (Fura-2AM; 5 mM; Calbiochem, Darmstadt, Germany). After three washes with artificial cerebrospinal fluid (aCSF: NaCl 125 mM, KCl 3 mM, NaH_2_PO_4_ 1.25 mM, CaCl_2_ 2 mM, MgSO_4_ 2 mM, D(C)-glucose 10 mM and HEPES 10 mM; pH 7.4 adjusted with NaOH) cells were positioned on an inverted epifluorescent optics microscope (Axiovert 135TV, Zeiss, Germany) with a xenon lamp and band-pass filters of 340 and 380 nm wavelengths. Cells were continuously perfused with aCSF (with or without added compounds) at 1.5 ml/s and visualized with a 40x oil-immersion objective (Zeiss). The responses were recorded by a ratiometric method, in which image pairs were obtained every 250 ms by exciting the preparations at 340 and 380 nm, since Fura-2 has an absorbance at 340 nm if bound to calcium, and at 380 nm if not, while the emission wavelength was maintained at 510 nm. The magnitude of the changes in the emission fluorescence of Fura-2 was taken as a measure of the changes in intracellular Ca^2+^ concentration (Ca^2+^ T amplitude).

Excitation wavelengths were changed through a high-speed wavelength switcher, Lambda DG-4 (Sutter Instrument, Novato, CA, United States). An estimation of intracellular Ca^2+^ concentration was given by the ratio between the emissions derived from the two excitation wavelengths of Fura-2 (R340/380). All data were recorded by a cooled CCD camera (Photometrics CoolSNAP fx) and processed and analyzed by MetaFluor software (Universal Imaging, West Chester, PA, United States). During the first 5 min of the trial, baseline Ca^2+^ levels were established. Addition of glutamate, as the stimulating agent (100 μM), was performed after those first 5 min corresponding to the baseline, and the cell response recorded in the next 10 min, this completing 15 min of total time recording. Representative videos of intracellular Ca^2+^ changes were obtained with the MetaFluor Analyst and using AVI Creator and Movie Maker software programs.

The frequency of Ca^2+^ transients was determined with MATLAB and Statistics Toolbox Release 2016a, The MathWorks, Inc. (Natick, MA, United States), as previously described (Martins et al., 2020). In the first 5 min of the experiment, a baseline was obtained. Mean and standard error of the mean (SEM) of all data recorded by each cell were obtained.

Ca^2+^ transient was considered valid when fura-2 excitation wavelength at 340 nm and 380 nm (F340/380) ratio was higher than the mean of baseline values plus 5 times the SEM. In addition, the normalized ratio was considered when above this margin for more than 5 s. For every region of interest, the peak of each transient, as well as the occurrence of transients, were recorded.

### Proteomics

#### Mass Spectrometric (MS) Analysis

The following cellular pellets were collected for proteomic analysis: (i) mSOD1 astrocytes, transfected or not with pre-miR-146a; (ii) naïve microglia; and (iii) WT motor neurons after incubation with mSOD1 astrocytic secretome (± pre-miR-146a). The cellular pellets were collected by trypsinization for 5 min, washed with PBS and immediately stored at −80°C. Each sample resulted from a pool of three biological replicates (cells isolated from different mice that were pooled down and sent for analysis at EMBL Proteomics Core Facility in Heidelberg, Germany. Samples were subjected to an in-solution tryptic digest using a modified version of the Single-Pot Solid-Phase-enhanced Sample Preparation (SP3) protocol (Moggridge et al., 2018). To this end, samples were added to Sera-Mag Beads (Thermo Scientific) in 10 μl 15% formic acid and 30 μl of ethanol. Binding of proteins was achieved by shaking for 15 min at room temperature. SDS was removed by 4 subsequent washes with 200 μl of 70% ethanol. Proteins were digested overnight at room temperature with 0.4 μg of sequencing grade modified trypsin (Promega) in 40 μl Hepes/NaOH, pH 8.4 in the presence of 1.25 mM TCEP and 5 mM chloroacetamide (Sigma-Aldrich). Beads were separated, washed with 10 μl of an aqueous solution of 2% DMSO and the combined eluates were dried down. Peptides were reconstituted in 10 μl of H_2_O and reacted for 1 h at room temperature with 80 μg of TMT10plex (Thermo Scientific) (Moggridge et al., 2018) label reagent dissolved in 4 μl of acetonitrile. Then, they were reacted with TMT-labeling reagent. 5% of each sample were mixed, purified by a reverse phase clean-up step (OASIS) and analyzed on a LUMOS system using a 1 h gradient. Calculated TMT ratios were used to adjust sample volumes to achieve a 1:1 ratio. The combined samples were subjected to a high pH offline fractionation yielding 12 fractions, each of those analyzed on a 2 h gradient on Orbitrap Fusion Lumos mass spectrometer (Thermo Scientific). Peptides were separated using an UltiMate 3000 nano RSLC system (Dionex) equipped with a trapping cartridge (Precolumn; C18 PepMap 100, 5 mm, 300 μm i.d. ×5 μm, 100 A°) and an analytical column (Acclaim PepMap 100. 75 cm × 50 cm C18, 3 mm, 100 Å) connected to a nanospray-Flex ion source. The peptides were loaded onto the trap column at 30 μl per min using solvent A (0.1% formic acid) and eluted using a gradient from 2 to 40% Solvent B (0.1% formic acid in acetonitrile) over 2 h at 0.3 μl per min. The Orbitrap Fusion Lumos was operated in positive ion mode with a spray voltage of 2.4 kV and capillary temperature of 275°C to analyze the peptides. MS spectra with a mass range of 375–1.500 m/z were acquired in profile mode using a resolution of 120.000 [maximum fill time of 50 ms or a maximum of 4 × 10^5^ ions (automatic gain control, AGC)]. Fragmentation was triggered for 3 s cycle time for peptide like features with charge states of 2–7 on the MS scan (data-dependent acquisition). Precursors were isolated using the quadrupole with a window of 0.7 m/z and fragmented with a normalized collision energy of 38. Fragment mass spectra were acquired in profile mode and a resolution of 30,000 in profile mode. Maximum fill time was set to 64 ms or an AGC target of 1e5 ions). The dynamic exclusion was set to 45 s.

#### Raw MS Data and Analysis

Acquired data were analyzed using IsobarQuant (Franken et al., 2015), Mascot V2.4 (Matrix Science) and a reverse UniProt FASTA Mus musculus (UP000000589) database, including common contaminants. The following modifications were considered: Carbamidomethyl (C, fixed), TMT10plex (K, fixed), Acetyl (N-term, variable), Oxidation (M, variable) and TMT10plex (N-term, variable). The mass error tolerance for full scan MS spectra was set to 10 ppm and for MS/MS spectra to 0.02 Da. A maximum of 2 missed cleavages were allowed. A minimum of 2 unique peptides with a peptide length of at least seven amino acids and a false discovery rate below 0.01 was required on the peptide and protein level (Savitski et al., 2015). 9083 proteins were identified, from which 6748 proteins were quantified. The raw output files of IsobarQuant were processed using the R programming language (R Development Core Team, 2020). As a quality filter, we only considered proteins that were quantified with at least two unique peptides. Raw TMT reporter ion intensities [Average expression (signal_sum)] were normalized using variance stabilization normalization (Huber et al., 2002). Differential expression was evaluated by computing the respective ratio of normalized TMT signals (additional parameters are indicted in Supplementary Tables 4–6). The proteins identified as hits were then classified by their link to biological processes using Gene Ontology (GO) annotation in Scaffold. Bioinformatic analysis of protein molecular function was done by using PANTHER Classification System (Version 16.0, released 2020-12-01) (Mi et al., 2021). Functional classification of gene lists for Mus musculus were examined using PANTHER Protein Class ontologies. MS proteomics data have been deposited to the ProteomeXchange Consortium via the PRIDE database^1^ (Perez-Riverol et al., 2019) partner repository with the dataset identifier PXD024294.

### Statistical Analysis

Results of at least three independent experiments were expressed as mean values ± SEM. Results of untreated or treated mSOD1 astrocytes, astrocyte-derived secretome and astrocyte-derived sEVs were related to respective untreated WT samples. Since we always compared at least three groups, we determined the differences between them by using one-way ANOVA followed by Bonferroni *post hoc* test. Statistical analysis was performed using GraphPad PRISM 7.0 (GraphPad Software, San Diego, CA, United States) *p* < 0.05 was considered as statistically significant.

## Results

### VS Is More Competent Than GUDCA in Restoring miR-146a Expression and Counteracting mSOD1 Astrocytic Aberrancies

In the present study, we first assessed whether our promising immunomodulators GUDCA and VS were able to restore the normal levels of miR-146a in the cortical mSOD1 astrocytes isolated from the 7-day-old mSOD1 mice and cultured for 13 DIV. Such miR-146a was found downregulated in cells presenting decreased gene and protein levels of GFAP, and upregulated gene expression of vimentin, Cx43, S100B and HMGB1 (Gomes et al., 2019). This miR-146a was found to fine tune Toll-like receptors and cytokine signaling (Taganov et al., 2006) and to be a key regulator of astrocyte-mediated inflammatory response (Iyer et al., 2012). Though miR-146 has at least 488 predicted targets, as reviewed in Brites (2020), we decided to investigate whether miR-146a inhibited two of its recognized target genes, the interleukin-1 receptor-associated kinase (IRAK) 1 and TNF receptor-associated factor (TRAF) 6 (Iyer et al., 2012; Saba et al., 2014). Actually, we have found that both *Irak1* and *Traf6* transcripts were upregulated in mSOD1 cortical astrocytes (Gomes et al., 2019).

**FIGURE 1:**
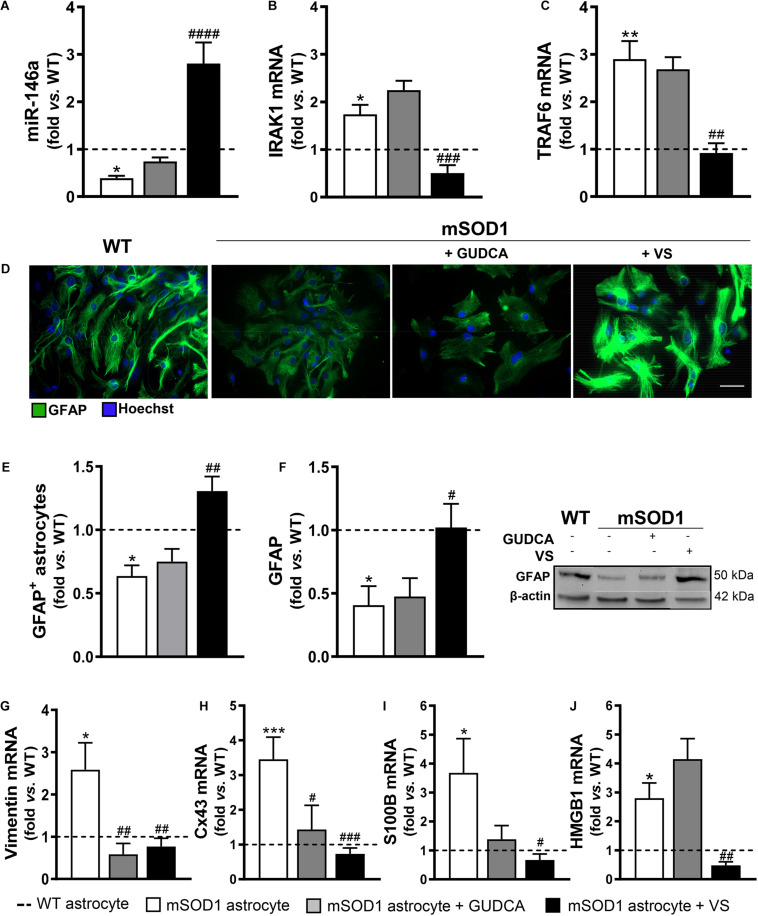
mSOD1 astrocytic aberrancies are more efficiently counteracted by VS than by GUDCA. Astrocytes were isolated from the cortex of SOD1-G93A (mSOD1) and wild type (WT) mice pups at 7-day-old and cultured for 13 days *in vitro*. Treatment with glycoursodeoxycholic acid (GUDCA) or dipeptidyl vinyl sulfone (VS) was performed in mSOD1 astrocytes. RT-qPCR analysis of **(A)** miRNA(miR)-146a, **(B)** interleukin-1 receptor associated kinase-1 (IRAK1) and **(C)** TNF receptor associated factor 6 (TRAF6), **(G)** vimentin, **(H)** connexin-43 (Cx43), **(I)** S100 calcium-binding protein B (S100B) and **(J)** high mobility group box 1 (HMGB1) was performed. **(D)** Representative images of astrocytes stained with glial fibrillary acidic protein (GFAP, in green) by immunocytochemistry and **(E)** quantification of GFAP-positive cells. Cell nuclei were stained with Hoechst dye (blue). **(F)** GFAP performed by Western blot analysis and the representative results from one blot are shown. Expression of β-actin was used as an endogenous control for Western Blot and RT-qPCR assays. SNORD110 was used as a reference gene for **(A)** analysis. Results are mean (±SEM) fold change *vs.* WT astrocytes from at least four independent experiments. *p < 0.05, **p < 0.01, and ***p < 0.001 *vs*. WT astrocytes; ^#^*p* < 0.05, ^##^*p* < 0.01, ^###^*p* < 0.001, and ^####^*p* < 0.0001 *vs*. untreated mSOD1 astrocytes. One-way ANOVA followed by Bonferroni *post hoc* test was used. Scale bar represents 20 μm.

We confirmed that miR-146a was indeed downregulated in the mSOD1 cortical astrocytes (*p* < 0.05), while their target genes IRAK1 and TRAF6 were upregulated (Figures 1A–C, at least *p* < 0.05). We also validated that the expression of the aberrant-associated genes was deregulated (Figures 1D–J, at least *p* < 0.05), attesting the characteristic phenotype previously established (Gomes et al., 2019). From the treatment of cells with either GUDCA or VS, only the later showed to effectively upregulate miR-146a and downregulate IRAK1 and TRAF6 (Figures 1A–C, at least *p* < 0.01), as well as to restore GFAP protein levels (Figures 1E,F). Interestingly, both GUDCA and VS abrogated the increased levels of vimentin and Cx43 (Figures 1G,H, at least *p* < 0.05), and were not toxic to the cells through the induction of early or late apoptosis, as depicted in Supplementary Figure 1. VS was again the most efficient in downregulating S100B and HMGB1 gene expression toward the WT levels (Figures 1I,J at least *p* < 0.05). Thus, VS demonstrated to have a broader reparative ability than GUDCA in recovering miR-146a expression and in counteracting the mSOD1 astrocytes associated reactive markers.

### Treatment of mSOD1 Astrocytes With Pre-miR-146a Abolishes Their Phenotypic Aberrancies

To assess whether miR-146a downregulation in cortical astrocytes isolated from mSOD1 pups was a player in the deregulated astrocyte polarization, we decided to transfect the cells with pre-miR-146a, as indicated in Material and Methods section, and to evaluate the post-treatment signature of the cells.

**FIGURE 2:**
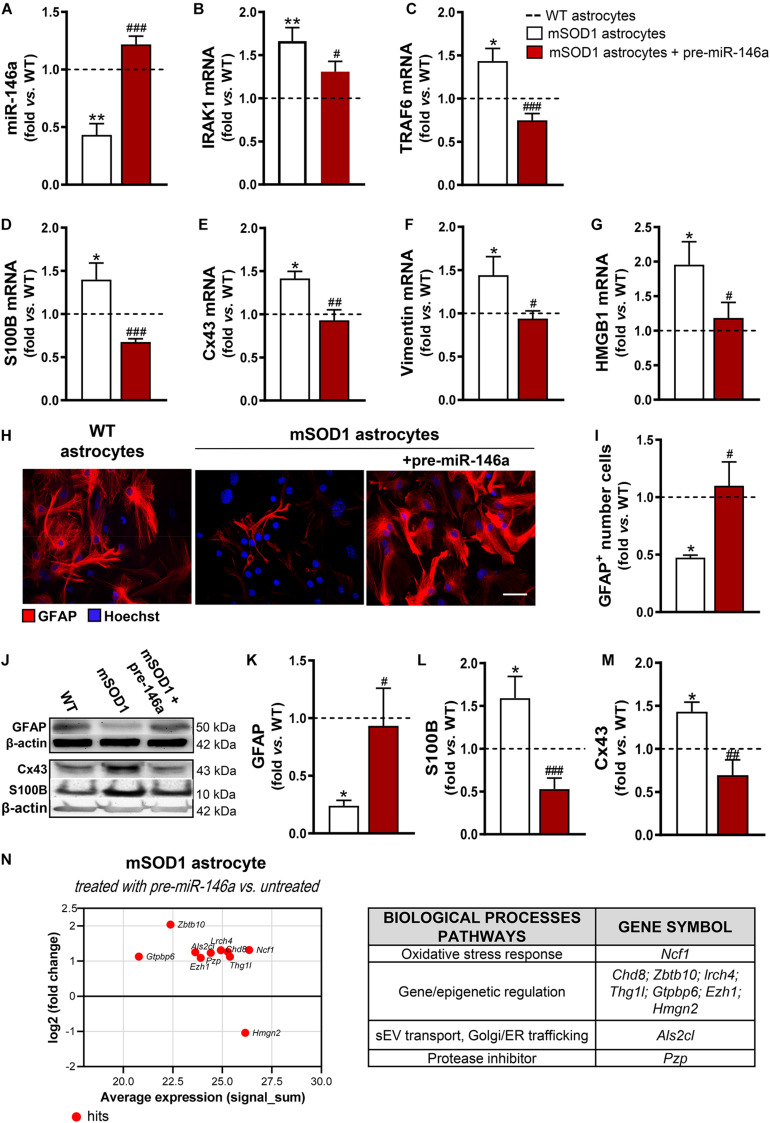
miR-146a upregulation successfully recovers GFAP expression and counteracts deregulated reactive markers in mSOD1 astrocytes. Astrocytes were isolated from the cortex of SOD1-G93A (mSOD1) and wild type (WT) mice with 7-day-old and cultured for 13 days *in vitro*. Transfection with pre-miR-146a was performed in mSOD1 astrocytes. RT-qPCR analysis of **(A)** miRNA(miR)-146a, **(B)** interleukin-1 receptor associated kinase-1 (IRAK1) and **(C)** TNF receptor associated factor 6 (TRAF6), **(D)** S100 calcium-binding protein B (S100B), **(E)** connexin-43 (Cx43), **(F)** vimentin, and **(G)** high mobility group box 1 (HMGB1) was performed. **(H)** Representative images of astrocytes stained with glial fibrillary acidic protein (GFAP, in red) by immunocytochemistry and **(I)** quantification of GFAP-positive cells. Cell nuclei were stained with Hoechst dye (blue). Protein expression of **(K)** GFAP, **(L)** S100B, and **(M)** Cx43 was performed by Western blot analysis and **(J)** representative results from one blot are shown. Expression of β-actin was used as an endogenous control for Western Blot and RT-qPCR assays. SNORD110 was used as reference gene for **(A)** analysis. Results are mean (±SEM) fold change *vs.* WT astrocytes from at least four independent experiments. *p < 0.05 and **p < 0.01 *vs*. WT astrocytes; ^#^*p* < 0.05, ^##^*p* < 0.01, and ^###^*p* < 0.001 *vs*. untreated mSOD1 astrocytes. One-way ANOVA followed by Bonferroni *post hoc* test was used. Scale bar represents 20 μm. **(N)** MA plot (left) was obtained by comparison of the proteomic profiles of pre-miR-146a treated mSOD1 astrocytes and the untreated ones. Here, *x* axis is the mean log2 intensity (fold change) of each protein between two profiles (A-value) and *y* axis is the average expression (signal_sum) of protein intensities (*M*-value). Summary tables (right panels) show the hits and their classification according to biological processes using PANTHER Classification System, as indicated in methods.

We noticed that the mSOD1 astrocytes were more sensitive to the transfection process than the WT cells, as judged by the loss of viable cells (*p* < 0.05) due to an increase of cell late apoptosis (*p* < 0.01) (Supplementary Figure 2). Interestingly, such features disappeared in cells transfected with pre-miR-146a, reinforcing the regulatory role of miR-146a in stress/immune-associated stimuli. A 3-fold increase in the levels of miR-146a in the mSOD1 astrocytes was achieved by the transfection with the pre-miR-146a, with values close to those presented by WT astrocytes (Figure 2A, *p* < 0.001), and its target genes IRAK1 (Figure 2B, *p* < 0.05) and TRAF6 (Figure 2C, *p* < 0.001) were reduced near to normal values.

Then, we investigated if the restoration of miR-146a levels was able to attenuate the aberrant phenotype of mSOD1 astrocytes. We verified that such treatment not only restored the gene control values of S100B (Figure 2D, *p* < 0.001), Cx43 (Figure 2E, *p* < 0.01), vimentin (Figure 2F, *p* < 0.05), and HMGB1 (Figure 2G, *p* < 0.05), but also recovered the GFAP protein expression levels (Figures 2H–K, *p* < 0.05). Besides, genetic upregulation of miR-146a restored the protein levels of S100B (Figures 2J,L, *p* < 0.001) and of Cx43 (Figures 2J,M, *p* < 0.01) toward the control values, attesting that the reduction of miR-146a expression may contribute to the cortical astrocyte aberrancies associated to the ALS disease and to MN degeneration.

To better understand the upregulation of miR-146a in the attenuation of ALS astrocyte-aberrancies and its potential targets we performed proteomic analysis. Data identified 10 hits after the treatment of mSOD1 astrocytes with pre-miR-146a *vs.* the untreated cells (Figure 2N and Supplementary Table 4). The increase of the neutrophil cytosol factor 1 encoded by the gene *Ncf1* may prevent the immune response of mSOD1 challenged astrocytes (Holmdahl et al., 2016; Zhao et al., 2017). In the same way, the upregulated zinc finger and BTB domain-containing 10, encoded by *Zbt10*, and the downregulated non-histone chromosomal protein HMG-17, encoded by *Hmgn2*, may turn the neurotoxic into a neuroprotective astrocyte phenotype. Increased expression of proteins encoded by the genes *Chd8, Lrch4, Thg1l, Gtpbp6*, and *Ezh1*, involved in gene transcription and epigenetic modifications, suggest a role of miR-146a in the accurate control of stage-specific gene activities required for proper astrocyte function. Upregulation of the pregnancy zinc protein, encoded by *Pzp* and of the guanine nucleotide exchange factor for Rab5 (the homolog *Als2c*l) by pre-miR-146a may contribute to prevent mSOD1 pathology, since the first inhibits the aggregation of misfolded proteins (Cater et al., 2019) and the later controls endosome dynamics, thus favoring mSOD1 clearance by sEVs (Hessvik and Llorente, 2018) and autophagy (Otomo et al., 2012).

Overall, our data highlight miR-146a upregulation as a promising therapeutic strategy to restore the steady-state profile of ALS astrocytes and suggest a new mechanism of action of VS as upregulating miR-146a in defective mSOD1 cortical astrocytes.

### Anti-miR-146a Transfection in WT Astrocytes Reproduces mSOD1 Cortical Astrocyte Aberrancies, Which Are Prevented by VS

**FIGURE 3:**
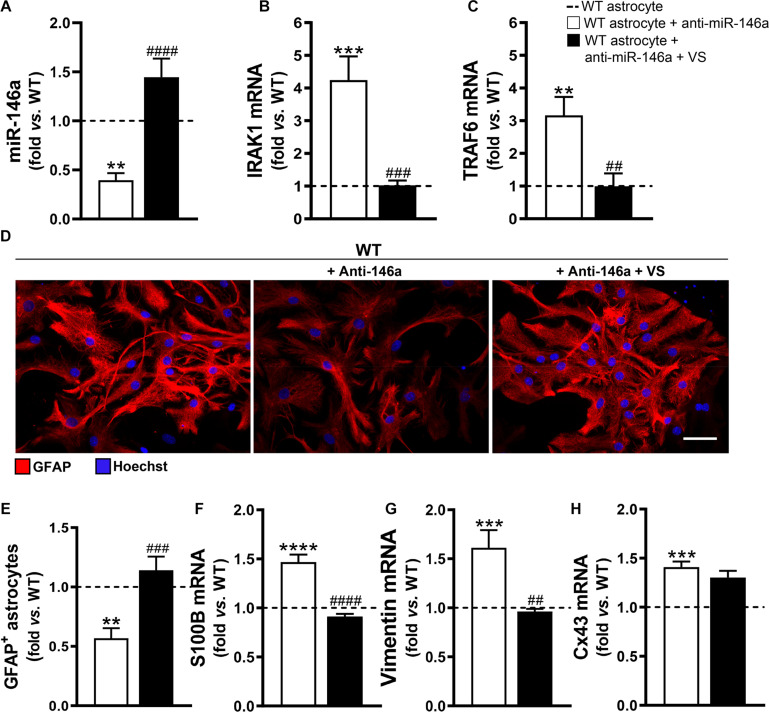
VS rescues GFAP levels and abolishes vimentin- and S100B-associated reactivity induced by the miR-146a inhibitor in WT astrocytes. Astrocytes were isolated from the cortex of wild type (WT) mice with 7-day-old and cultured for 13 days *in vitro*. Transfection with anti-miR-146a followed by treatment with dipeptidyl vinyl sulfone (VS) was performed in these cells. RT-qPCR analysis of **(A)** miRNA(miR)-146a, **(B)** interleukin-1 receptor associated kinase-1 (IRAK1), **(C)** TNF receptor associated factor 6 (TRAF6), **(F)** S100 calcium-binding protein B (S100B), **(G)** vimentin and **(H)** connexin-43 (Cx43) was performed. SNORD110 was used as reference gene for **(A)** analysis and β-actin for **(B,C,F–H)** analysis. **(D)** Representative images of astrocytes stained with glial fibrillary acidic protein (GFAP, red) in red by immunocytochemistry and **(E)** respective quantification of the GFAP-positive cells. Cell nuclei were stained with Hoechst dye (blue). Results are mean (±SEM) fold change *vs.* untreated WT astrocytes from at least three independent experiments. **p < 0.01, ***p < 0.001, and ****p < 0.0001 *vs*. untreated WT astrocytes; ^##^*p* < 0.01, ^###^*p* < 0.001, and ^####^*p* < 0.0001 *vs*. WT astrocytes treated with anti-miR-146a. One-way ANOVA followed by Bonferroni *post hoc* test was used. Scale bar represents 20 μm.

To explore if the efficacy of VS in counteracting the aberrant profile of astrocytes was mediated through its direct action on miR-146a, we downregulated miR-146a expression in astrocytes isolated from the brain cortex of B6SJLF1/J non-transgenic WT mice. We confirmed that the transfection of cortical astrocytes with anti-miR-146a, after being isolated from 7-day-old WT mice and cultured for 13 days, led to a 2-fold decrease in the expression of miR-146a (Figure 3A, *p* < 0.01). In conformity, IRAK1 and TRAF6 gene expression levels were overexpressed (Figures 3B,C). Such immune deregulation was translated into the typical astrocyte ALS fingerprint, i.e., low GFAP protein and increased S100B, vimentin and Cx43 mRNAs (Figures 3D–H), as observed in mSOD1 cortical astrocytes.

Then, we wonder whether VS would be able to invert the signature of ALS cells produced by miR-146a inhibition. Addition of VS was in fact able to restore the WT astrocyte profile even after the anti-miR-146a transfection. Indeed, WT astrocytes co-treated with VS and anti-miR-146a presented the steady-state signature of healthy cells for miR-146a and its targets IRAK1 and TRAF6 gene expression levels (Figures 3A–C), as well as for GFAP, S100B and vimentin (Figures 3D–G). Interestingly, VS was not as effective as pre-miR-146a in abrogating the elevated levels of Cx43 derived from the anti-miR-146a treatment in WT astrocytes.

Data suggest that miR-146a downregulation is associated with a pathological ALS astrocyte signature, which can be rescued through immunomodulation with pre-miR-146 or by addition of VS, reinforcing depleted miR-146a as a promising target for the restoration of cell homeostasis in the brain cortex.

### VS Counteracts High Intracellular Ca^2+^ in mSOD1 Astrocytes, but Only Pre-miR-146a Prevents Enhanced Frequency and Amplitude of Glutamate-Induced Ca^2+^ Transients

Among the several toxic factors secreted by mSOD1 astrocytes is the excess of Ca^2+^ release that was found to contribute to the pathogenesis of ALS (Kawamata et al., 2014). Though that Ca^2+^ signals are fundamental for the regulation of intracellular signaling, as well as for vesicular secretion processes in astrocytes, their acute and chronic changes in response to brain injury were associated to dysregulated astrocyte-neuron communication (Vardjan et al., 2017). Evidence showed that aberrant Ca^2+^ signals in reactive astrocytes are implicated in the onset, progression and severity of neurodegenerative diseases (Shigetomi et al., 2019). Here, we intended to explore the intracellular calcium dysregulation in ALS astrocytes and the ability of pre-miR-146a and VS to prevent such abnormality and restore the neuroprotective profile of astrocytes.

We evaluated intracellular Ca^2+^ dynamics by live-cell imaging using the fluorescent dye Fura-2. Ca^2+^ signaling was measured without any stimulation (baseline) or in response to glutamate addition, which is a trigger of free cytoplasmic Ca^2+^ oscillations (Cornell-Bell et al., 1990) and known to be extracellularly elevated in ALS (Yamanaka and Komine, 2018).

**FIGURE 4:**
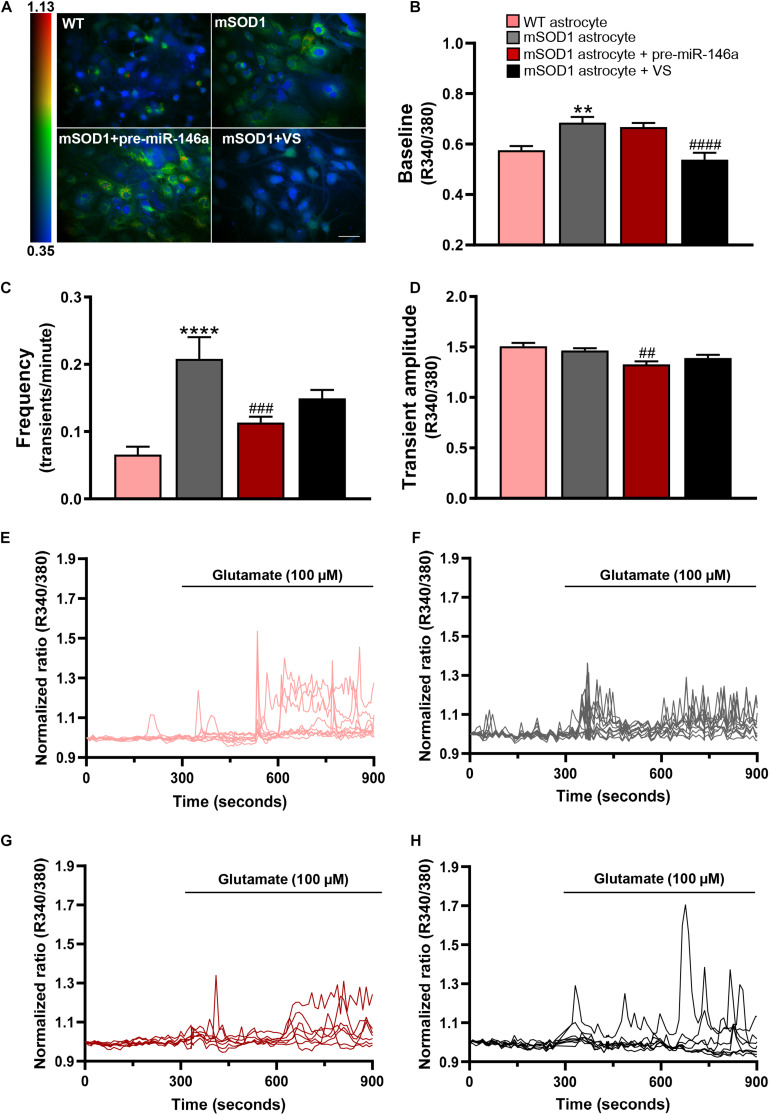
VS reduces the upregulated intracellular Ca^2+^, while pre-miR-146a normalizes the number and amplitude of glutamate-induced Ca^2+^ transients in mSOD1 astrocytes. Astrocytes were isolated from the cortex of SOD1-G93A (mSOD1) and wild type (WT) mice with 7-day-old and cultured for 13 days *in vitro*. Transfection with pre-miR-146a or treatment with dipeptidyl vinyl sulfone (VS) was performed in mSOD1 astrocytes. Cells were incubated at 37°C for 45 min with the calcium (Ca^2+^) sensitive fluorescent dye fura-2 acetoxymethyl ester (Fura-2), followed by glutamate addition (100 μM). **(A)** Pseudocolored Fluo4 fluorescence images in WT, untreated and treated-mSOD1 astrocytes show a prominent rise in intracellular Ca^2+^ and respective **(B)** changes in the baseline of Fura-2 fluorescence. The color code refers to the fluorescence ratio 340 nm/380 nm, with higher ratio reflecting higher intracellular Ca^2+^. **(C)** Summary plot of the frequency of transients per minute and **(D)** the amplitude of the Ca^2+^ responses. Results are mean (±SEM) from at least 40 responsive cells from six independent experiments. Representative profiles of normalized Ca^2+^ responses of at least 8 cells in **(E)** WT astrocytes and **(F)** mSOD1 astrocytes treated with **(G)** pre-miR-146a and **(H)** VS. The first 300 s represents the changes of Fura-2 fluorescence in the baseline. The remaining 600 s refers to changes after glutamate addition. **p < 0.01 and ****p < 0.0001 *vs.* WT astrocytes; ^##^*p* < 0.01, ^###^*p* < 0.001, and ^####^*p* < 0.0001 *vs.* untreated mSOD1 astrocytes. One-way ANOVA followed by Bonferroni *post hoc* test was used. Scale bar represents 20 μm.

Ca^2+^ signaling was assessed by the fluorescence intensity response of Fura-2 after consecutive stimulation at 340 and 380 nm and quantified in individual cells the ratio between the two responses (R340/380) (Figure 4 and Supplementary Videos 1–4). mSOD1 astrocytes revealed a significant increase of the basal Fura-2 340/380 ratio in (*p* < 0.01) when compared with matched *vs.* WT astrocytes (Figures 4A,B,E,F and Supplementary Videos 1, 2). Treatment with VS restored the WT values (*p* < 0.0001), but not pre-miR-146a upregulation (Figures 4A,B,G,H and Supplementary Videos 3, 4, respectively).

Treatment of astrocytes with 100 μM glutamate produced a higher frequency of Ca^2+^ transients in mSOD1 astrocytes, as compared with the WT astrocytes (Figures 4C,E,F, *p* < 0.0001 and Supplementary Videos 5, 6). In the pre-miR-146a transfected mSOD1 astrocytes the addition of glutamate prevented the increased number of transient Ca^2+^ signaling events observed in the non-modulated cells (Figures 4C,G, *p* < 0.001 and Supplementary Video 7). A similar finding was observed in the amplitude of Ca^2+^ waves, where a small, but significant decrease occurred in glutamate-treated mSOD1 astrocytes previously transfected with pre-miR-146a, but not with VS (Figures 4D,H, *p* < 0.01 and Supplementary Video 8), supporting a delayed response of Ca^2+^ release in the mSOD1 astrocytes upregulated for miR-146a.

Results suggest that VS and pre-miR-146a differently regulate intracellular Ca^2+^ load. In the absence of increased extracellular glutamate, VS is a more promising strategy in sustaining intracellular Ca^2+^ homeostasis. However, if excitotoxity is predicted to occur, then pre-miR-146a treatment has advantage over the use of VS to control Ca^2+^ dynamic dysregulation.

### Overexpression of Pre-miR-146a in mSOD1 Astrocytes Is Recapitulated in Their Derived sEVs

Besides the paracrine influence of the Ca^2+^ signals derived from reactive astrocytes, it is currently accepted that other components of the astrocytic secretome contribute to neurotoxicity and microglia activation in ALS, mainly due to astrocytic-derived sEVs and their content in miRNAs and misfolded/mutated proteins (Silverman et al., 2016; Ferrara et al., 2018; Varcianna et al., 2019). Recently, we have shown that astrocytes isolated from the SC and the brain cortex of mSOD1 mouse pups are depleted in miR-146a, miR-155 and miR-21 (Gomes et al., 2020).

**FIGURE 5:**
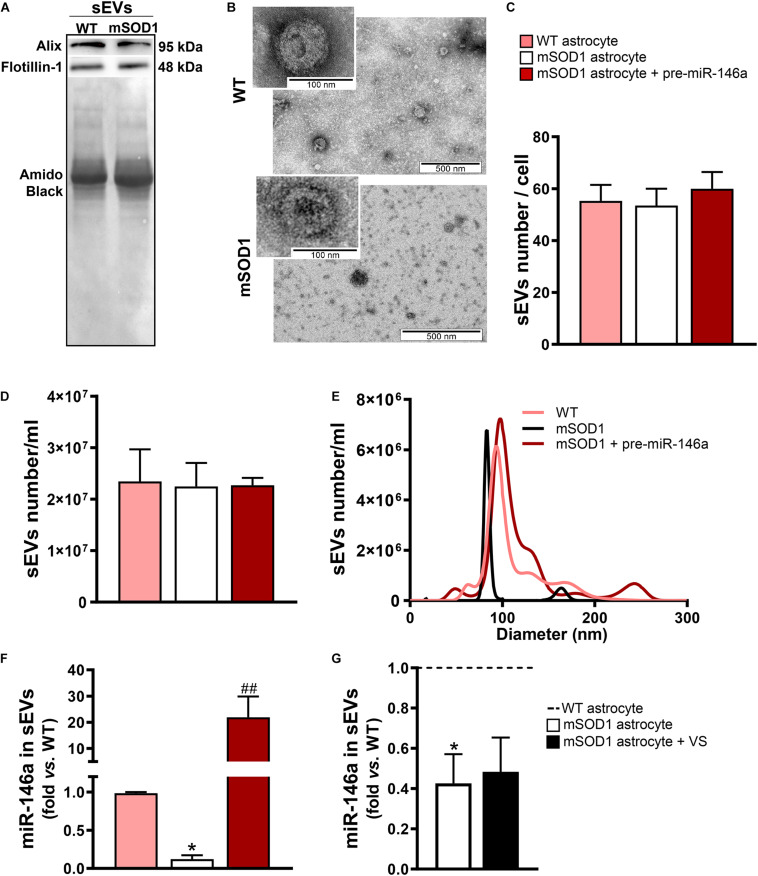
sEVs derived from pre-miR-146a-treated mSOD1 astrocytes show enriched content in miR-146a, but not those from VS-treated cells. Astrocytes were isolated from the cortex of SOD1-G93A (mSOD1) and wild type (WT) mice pups at 7-day-old and cultured for 13 days *in vitro*. Transfection with pre-miR-146a and treatment with dipeptidyl vinyl sulfone (VS) were performed in mSOD1 astrocytes. Small extracellular vesicles (sEVs) were isolated from the secretome of astrocytes by differential ultracentrifugation. **(A)** Results from one blot shows the expression of sEV markers (Alix and Flotillin-1). **(B)** Representative images obtained by transmission electron microscopy of sEVs show their cup shape morphology. Results from **(C)** number of sEVs per cell, **(D)** concentration (sEVs number/mL), and **(E)** size distribution derived from Nanoparticle Tracking Analysis using NanoSight. **(F,G)** RT-qPCR analysis of miRNA(miR)-146a expression in sEVs was performed. Spike and SNORD were used as endogenous controls. Results are mean (±SEM) fold change *vs.* sEVs-derived WT astrocytes from at least three independent experiments. *p < 0.05 *vs*. sEVs from WT astrocytes; ^##^*p* < 0.01 *vs*. sEVs from untreated mSOD1 astrocytes. One-way ANOVA followed by Bonferroni *post hoc* test was used.

In this sense, we next assessed if the sEVs isolated from the mSOD1 astrocyte were depleted in miR-146a and whether the treatment of the cells with pre-miR-146a or VS was able to enrich the sEVs in such miRNA. We first characterized the sEVs for the presence of characteristic proteins such as Alix and Flotillin-1 (Figure 5A) and confirmed their cup-shape morphology by transmission electron microscopy (TEM) (Figure 5B), proving the efficiency of sEV isolation. Next, we attested that the transfection of the astrocytes with pre-miR-146 did not modified the number, size, or the concentration of sEVs (Figures 5C–E), relatively to those from WT and mSOD1 astrocytes. Interestingly, when assessed for miR-146a cargo, we observed a 22-fold increase of miR-146a in sEVs derived from the pre-miR-146a-treated astrocytes (Figure 5F, *p* < 0.01). In contrast, despite the ability of VS to increase the intracellular levels of miR-146a in the depleted mSOD1 astrocytes, it was unable to induce its integration in the secreted sEVs from VS-treated astrocytes, as compared with the untreated ones (Figure 5G).

From the data obtained we may conclude that only pre-miR-146a treatment of mSOD1 astrocytes has beneficial consequences in the active transfer of this miRNA into sEVs, thus re-establishing paracrine signaling to recipient cells, and effects in their target genes. We cannot, however, disregard that VS can also have positive influence on the neighboring cells through astrocyte-derived unidentified soluble factors.

### Neuroprotective Strategies of mSOD1 Astrocytes by Pre-miR-146a or VS Immunomodulation Involve Distinct Signaling Pathways

Once mSOD1 astrocytes were shown to release neurotoxic factors that lead to MN demise (Nagai et al., 2007; Gomes et al., 2019, 2020), we next decided to explore whether the effects produced by pre-miR-146a or VS modulation in the cells would translate into a more neuroprotective secretome.

**FIGURE 6:**
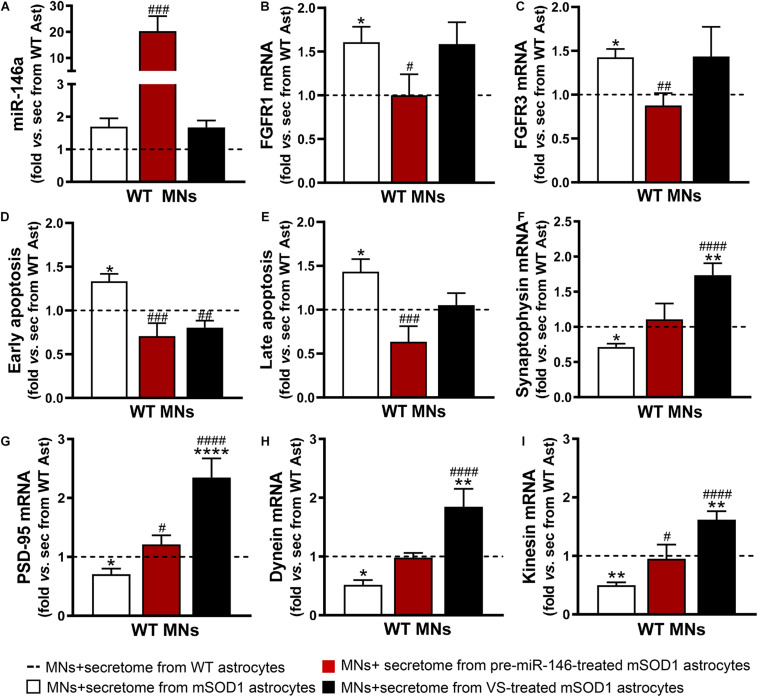
Both VS and pre-miR-146a treatment are effective in restoring the neuroprotective profile of mSOD1 astrocytes but reveal distinct benefits. Astrocytes (Ast) were isolated from the cortex of SOD1-G93A (mSOD1) and wild type (WT) mice with 7-day-old and cultured for 13 days *in vitro*. Transfection with pre-miR-146a or treatment with dipeptidyl vinyl sulfone (VS) was performed in mSOD1 astrocytes. Cell-derived secretome (sec) was incubated in WT NSC-34 motor neurons (MNs) for 48 h. Analysis of **(A)** miRNA(miR)-146a, **(B)** fibroblast growth factor receptor 1 (FGFR1), **(C)** FGFR3, **(F)** synaptophysin, **(G)** post-synaptic protein 95 (PSD95), **(H)** dynein, and **(I)** kinesin in WT MNs were assessed by RT-qPCR. SNORD110 was used as reference gene for **(A)** analysis and β-actin for **(B,C,F–I)** analysis. **(D)** Early apoptotic cells (annexin V-PE positive and 7-AA negative) and **(E)** late apoptotic/necrotic cells (annexin V-PE and 7-AA positive) were assessed by Guava Nexin^®^ Reagent in the WT MNs after secretome interaction. Results are mean (±SEM) fold change *vs.* MNs + secretome from WT astrocytes from at least three independent experiments. ^∗^*p* < 0.05, ^∗∗^*p* < 0.01, and **** *p* < 0.0001 *vs*. MNs + secretome from WT astrocytes; ^#^*p* < 0.05, ^##^*p* < 0.01, ^###^*p* < 0.001, and ^####^*p* < 0.0001 *vs*. MNs + secretome from mSOD1 astrocytes. One-way ANOVA followed by Bonferroni *post hoc* test was used.

To explore this issue, we used WT MNs given the reasons explained in the Materials and Methods section, including our expertise with these cells and previous data (Vaz et al., 2015; Pinto et al., 2017; Gomes et al., 2019). We observed that the increase of miR-146a in the WT MNs was only achieved by the secretome from the mSOD1 astrocytes overexpressing miR-146a (Figure 6A, *p* < 0.001). Despite the upregulation of miR-146a in the mSOD1 astrocytes treated with VS (Figure 1A), miR-146a was not enriched in sEVs isolated from the secretome of VS-treated mSOD1 astrocytes (Figure 5G), or in WT MNs treated with the secretome from VS-treated mSOD1 astrocytes (Figure 6A). These findings suggest that soluble miR-146a may have accounted for its rise in MNs, an issue that deserves future studies for clarification.

Expression of the fibroblast growth factor receptor 1 (FGFR1) and 3 (FGFR3) were found in neurons and associated to major roles during neurodevelopment, namely in dendritogenesis (Huang et al., 2017; Okada et al., 2019). We found that the secretome from mSOD1 astrocytes induced the overexpression of both receptors in MNs (Figures 6B,C). When modulated, while that from VS-treated mSOD1 astrocytes did not cause any alteration, the secretome from pre-miR-146a-treated mSOD1 astrocytes sustained their levels close to WT ones, a finding not achieved with VS. Transfection of mSOD1 astrocytes with pre-miR-146a also prevented MN death by early and late apoptosis processes (Figures 6D,E, *p* < 0.05) induced by the secretome from the non-modulated pathological cells. Only the early apoptosis was prevented by the secretome from mSOD1 astrocytes previously treated with VS (*p* < 0.01).

As depicted in Figures 6F–I, secretome from mSOD1 astrocytes decreased pre- and post-synaptic proteins (synaptophysin and PSD-95, respectively), as well as proteins involved in anterograde and retrograde axonal transport (kinesin and dynein, respectively) (*p* < 0.05 for all). In this case, major benefits were obtained by the VS treatment that markedly upregulated the expression of all these genes (*p* < 0.001), though the secretome from pre-miR-146a-treated mSOD1 astrocytes reversed the deficient PSD-95 gene expression and that of the anterograde transport motor kinesin (*p* < 0.05). To be sure that the effects observed in MNs were due to the secretome from the VS-treated astrocytes and not to the presence of the own compound in the secretome, we evaluated the expression of PSD-95, kinesin and miR-146a in MNs exposed to the media from untreated and VS-treated WT astrocytes. Results showed no differences between such conditions (Supplementary Figure 3). Overall, our findings evidence that the treatment of mSOD1 astrocytes with VS prevents the release of toxic factors involved in the loss of MN synaptic and axonal dynamics.

**FIGURE 7:**
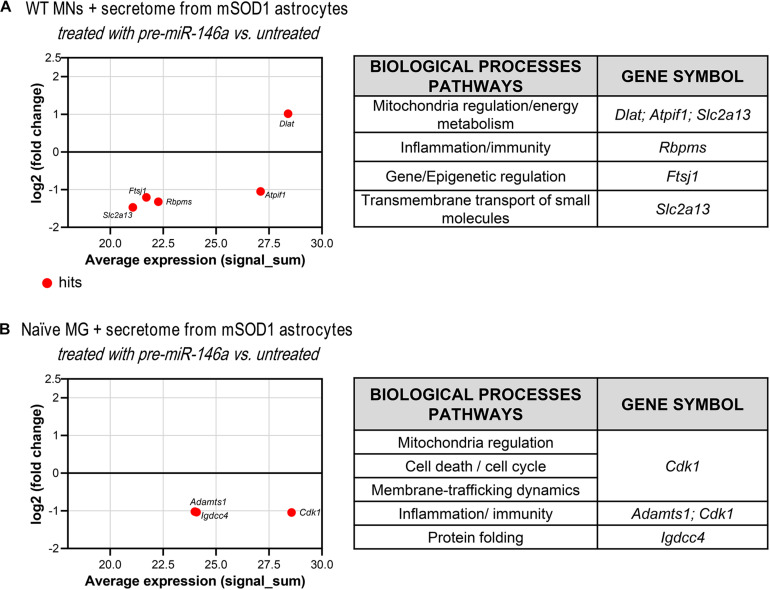
Proteomic analysis reveals that treatment of mSOD1 astrocytes with pre-miR-146a reverts MN dysfunction and microglia activation by paracrine mediators. Astrocytes were isolated from the cortex of SOD1-G93A (mSOD1) mice with 7-day-old and cultured for 13 days *in vitro*. Transfection with pre-miR-146a was performed. Cell-derived secretome was added to **(A)** WT NSC-34 motor neurons (MNs) for 48 h or **(B)** naïve N9 microglia for 24 h. MA plots (left panels) were obtained by comparison of the proteomic profiles of the treatment with the secretome of pre-miR-146a treated mSOD1 astrocytes and the untreated ones. Here, *x* axis is the mean log2 intensity (fold change) of each protein between two profiles (*A*-value) and *y* axis is the average expression (signal_sum) of protein intensities (*M*-value). Summary tables (right panels) show the obtained hits and their classification according to biological processes using PANTHER Classification System as indicated in methods.

Since we have observed an increase of miR-146a in the WT MNs treated with the secretome from the mSOD1 astrocytes transfected with pre-miR-46a, we next performed proteomic analysis to explore some of its potential targets in these cells and better understand related effects. We found one upregulated and 4 downregulated hits (Figure 7A and Supplementary Table 5). The first was associated to the induction of the pyruvate dehydrogenase complex encoded by *Dlat* (Goguet-Rubio et al., 2016) that enhances the production of ATP with benefits in preventing MN energetic stress that characterizes ALS (Vandoorne et al., 2018). To that it may have accounted the inhibition of the protein encoding for *Atpif1* that also favors the ATP hydrolysis and ameliorates ALS MN mitochondrial dysfunctionalities (Li et al., 2010; Chen et al., 2014). These potential benefits may be increasingly sustained by the downregulation of FtsJ RNA 2′-O-Methyltransferase 1 (*Ftsj1*), known to reduce apotosis (He et al., 2020). Another important potential target was the inhibition of the Solute Carrier Family 2 (Facilitated Glucose Transporter) Member 13 protein encoded by *Slc2a13*, recently identified a risk factor in frontotemporal dementia (Sirkis et al., 2019). Its silencing was shown to diminish APP processing (Teranishi et al., 2015) and may impact on the crosstalk between APP and mSOD1, which seems to contribute to ALS pathology (Rabinovich-Toidman et al., 2015). Also, adding to a better MN functionality is the decrease of RNA-binding protein with multiple splicing encoded by the *Rbpms* gene that favors synaptogenesis and may be associated with the increased PSD-95 that we observed (Hornberg et al., 2013).

These results clearly show distinct beneficial effects by the secretome from VS-treated astrocytes with protective properties on synaptic dynamics and axonal transport, while those depending on pre-miR-146a treated astrocytes were predominantly related with neuronal survival, miR-146a enrichment and regulation of FGFR1 and FGFR3. As far as we know, this is the first time that mSOD1 astrocytes are described as showing an elevation of such fibroblast growth factor receptors.

### Secretome From mSOD1 Astrocytes Transfected With Pre-miR-146a Shows to Better Preserve Microglia Healthy State Than the Treatment With VS

Besides affecting MNs, astrocytes also release soluble factors that influence microglia activation, but such signaling events in the context of ALS disease are still unclarified.

**FIGURE 8:**
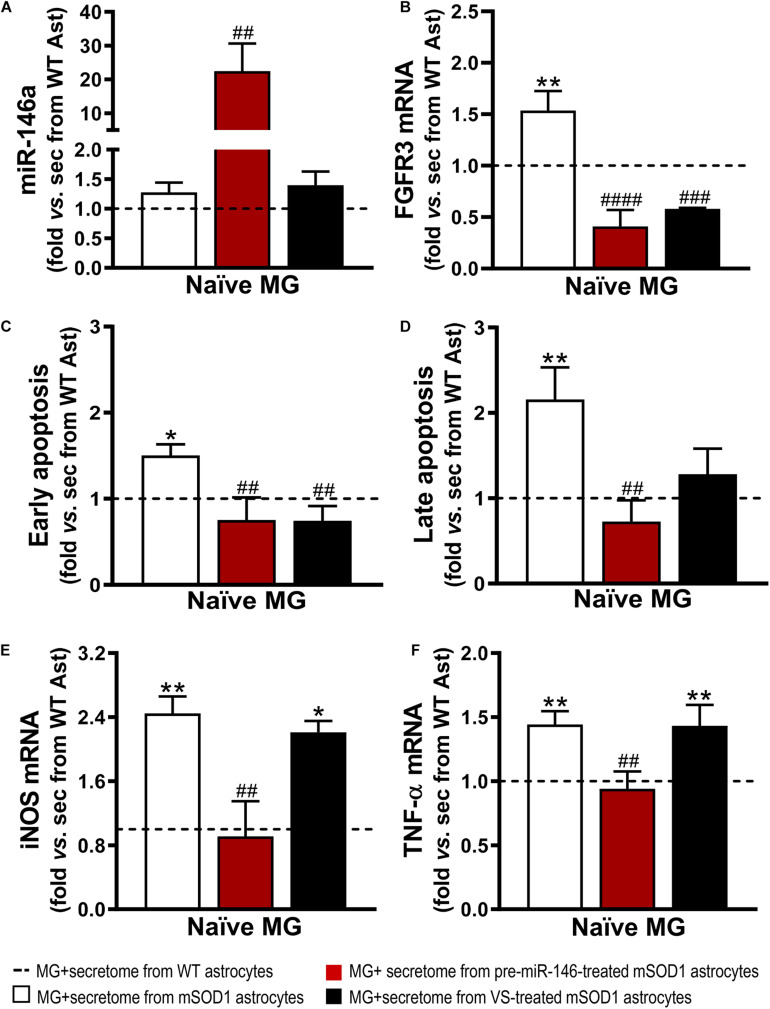
Secretome from pre-miR-146a-treated mSOD1 astrocytes translates into microglial miR-146 increase and regulation of cell activation by the untreated secretome, while also prevents cell demise similarly to VS modulation. Astrocytes (Ast) were isolated from the cortex of SOD1-G93A (mSOD1) and wild type (WT) mice with 7-day-old and cultured for 13 days *in vitro*. Transfection with pre-miR-146a or treatment with dipeptidyl vinyl sulfone (VS) was performed in mSOD1 astrocytes. Secretome was isolated and incubated in naïve N9 microglia for 24 h. Expression of **(A)** miRNA(miR)-146a, **(B)** fibroblast growth factor receptor 3 (FGFR3), **(E)** inducible nitric oxide synthase (iNOS) and **(F)** tumor necrosis alpha (TNF-α) in microglia was assessed by RT-qPCR. SNORD110 was used as reference gene for **(A)** analysis and β-actin for **(B,E,F)** analysis. **(C)** Early apoptotic (Annexin V-PE positive and 7-AAD negative), and **(D)** late apoptotic/necrotic cells (Annexin V-PE and 7-AAD positive) were assessed by Guava Nexin^®^ Reagent in the microglia after secretome interaction. Results are mean (±SEM) fold change *vs.* MG + secretome from WT astrocytes from at least three independent experiments. *p < 0.05 and **p < 0.01 *vs*. MG + secretome from WT astrocytes; ^##^*p* < 0.01, ^###^*p* < 0.001, and ^####^*p* < 0.0001 *vs*. MG + secretome from mSOD1 astrocytes. One-way ANOVA followed by Bonferroni *post hoc* test was used.

It is known that the expression of FGFR3 in microglia has been linked to enhanced microglial migration and phagocytosis of neuronal debris (Noda et al., 2014). However, inappropriate phagocytosis of live neurons and excessive neuronal loss, termed phagoptosis (Brown and Neher, 2014), may have detrimental consequences on the patient outcome. Similarly, to our findings on the effects produced by the secretome from mSOD1 astrocytes on WT MNs, naïve N9 microglial cells showed increased gene expression of FGFR3 (Figure 8B). Both VS and pre-miR-146a immunomodulation of mSOD1 astrocytes were effective in reducing such expression (*p* < 0.001). Further results showed that the secretome of mSOD1 astrocytes induced early and late apoptosis (Figures 8C,D, at least *p* < 0.05) that were prevented when the cells were treated with pre-miR-146a or VS (only early apoptosis). As expected, the secretome from mSOD1 astrocytes also activated and polarized microglia to overexpress iNOS (Figure 8E, *p* < 0.01) and TNF-α mRNAs (Figure 8F, *p* < 0.01). Only the treatment of mSOD1 astrocytes with pre-miR-146a showed to be efficient in counteracting the expression of such inflammatory mediators and in switching microglia into their steady-state (*p* < 0.01).

As before, and since we found an overexpressed miR-146a in the microglia treated with the secretome from pre-miR-146a mSOD1 astrocytes, we proceeded to proteomic analysis to investigate how the potential targets related with the improved effects we observed. Downregulation of the cyclin-dependent kinase 1 levels (encoded by *Cdk1)* may have contributed to the regulation of iNOS and TNF-α in the microglia treated with the secretome from mSOD1 astrocytes relatively to the untreated one (Figure 7B and Supplementary Table 6). Actually, increase of iNOS and TNF-α is associated to microglia activation and neuroinflammation (Sheng et al., 2011) and CDK inhibitors were shown to reduce them in several conditions (Leitch et al., 2009; Skovira et al., 2016). As for the decrease in the metalloproteinase with thrombospondin motifs 1, encoded by the gene *Adamts1*, it was suggested to be a modulator of the immune cell response in tumors (Rodriguez-Baena et al., 2018), and found elevated in LPS-activated microglia (Chiu et al., 2013). In what concerns the immunoglobulin superfamily DCC subclass member 4, encoded by *Igdcc4* and accepted as a biomarker of hepatocellular carcinoma (Zweerink et al., 2020) and of innate immune/inflammatory response (Sanz-Pamplona et al., 2014), it should be explored in the future as part of the recovering microglia steady-state profile by pre-miR-146a in ALS cortical astrocytes through paracrine signaling.

In sum, both pre-miR-146a and VS modulatory effects on ALS astrocytes prevented secretome-mediated microglial demise and FGFR3 increase. However, the transfection of the mSOD1 astrocytes with pre-miR-146a had a higher efficiency in preventing the microglial elevation of iNOS and TNF-α gene expression levels than the VS treatment. We next investigated if the effects observed on the microglia deactivation by the secretome from the mSOD1 astrocytes treated with pre-miR-146a were mediated by sEVs, previously shown to be enriched in this miRNA in the section “Overexpression of Pre-miR-146a in mSOD1 Astrocytes Is Recapitulated in Their Derived sEVs.”

### sEVs From mSOD1 Astrocytes Treated With Pre-miR-146a Are Preferentially Captured by Microglia, Enhancing Its Intracellular Upregulation, and Together With VS Counteract Cell Activation by the Pathological sEVs

Misfolded and mutated SOD1 were shown to be propagated intercellularly by sEV-dependent and independent mechanisms (Grad et al., 2014), and to have impact on astrocyte protein secretion pathways, thus accounting to MN pathology and dissemination (Basso et al., 2013). We have previously demonstrated that sEVs from NSC-34 hSOD1G93A MN-like cells cause alterations on N9 microglia polarization (Pinto et al., 2017). However, the effects produced by the secretome of pathological astrocytes on microglia polarization have been scarcely investigated.

**FIGURE 9:**
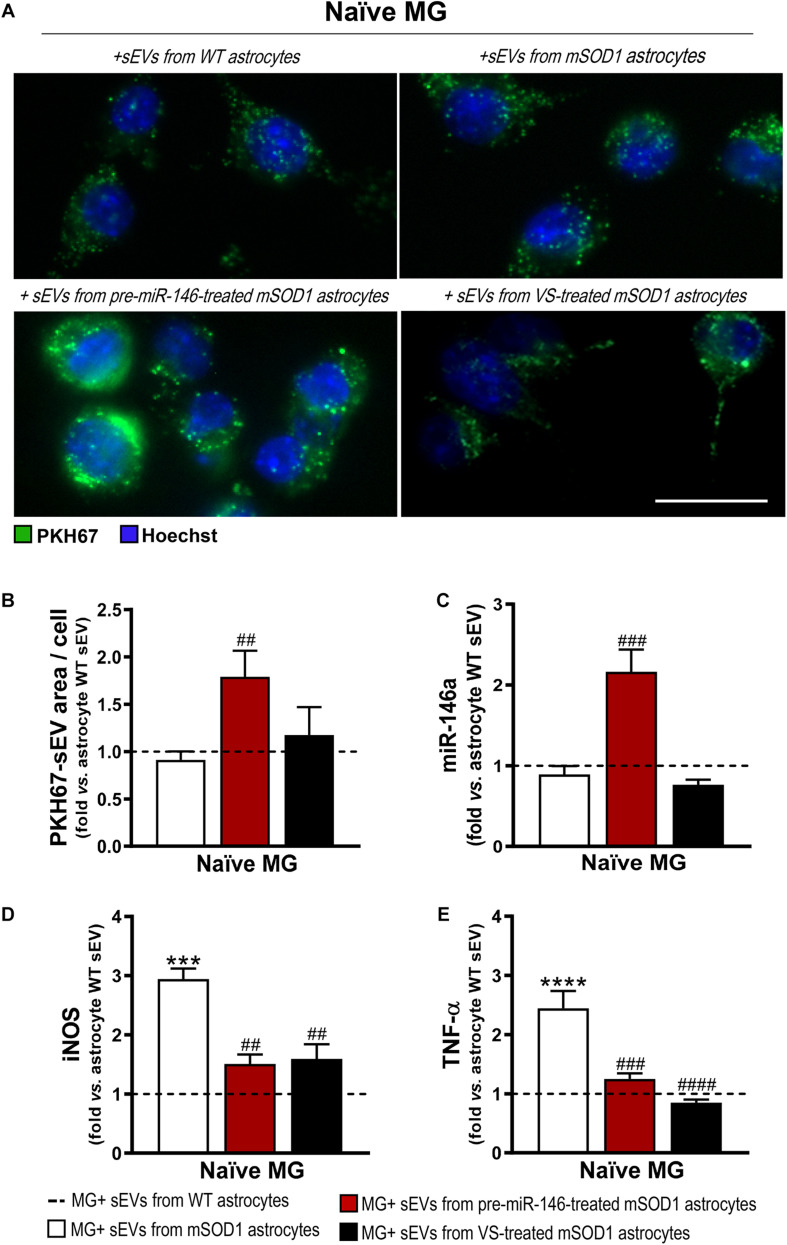
Microglia treated with sEVs from mSOD1 astrocytes modulated with pre-miR-146a or VS show different sEV internalization and miR-146a expression, but similar protection from activation by untreated-sEVs. Astrocytes were isolated from the cortex of SOD1-G93A (mSOD1) and wild type (WT) mice with 7-day-old and cultured for 13 days *in vitro*. Transfection with pre-miR-146a or VS treatment was performed in mSOD1 astrocytes. Small extracellular vesicles (sEVs) were isolated by differential ultracentrifugation and labeled with PKH67 cell linker, followed by incubation with naïve N9 microglia for 24 h. **(A)** Representative images of sEVs stained with PKH67 cell linker (green); **(B)** mean fluorescence area positive for PKH67-sEVs per cell. Cell nuclei were stained with Hoechst dye (blue). Expression of **(C)** miRNA(miR)-146a, **(D)** inducible nitric oxide synthase (iNOS) and **(E)** tumor necrosis alpha (TNF-α) in microglia was assessed by RT-qPCR. SNORD110 was used as reference gene for **(C)** analysis and β-actin for **(D–E)** analysis. Results are mean (±SEM) fold change *vs.* MG + sEVs from WT astrocytes from at least three independent experiments. ***p < 0.001 and ****p < 0.0001 *vs*. MG + sEVs from WT astrocytes; ^##^*p* < 0.01, ^###^*p* < 0.001, and ^####^*p* < 0.0001 *vs*. MG + sEVs from mSOD1 astrocytes. One-way ANOVA followed by Bonferroni *post hoc* test was used. Scale bar represents 20 μm.

Similarly to what we have previously observed for WT MNs, the secretome from pre-miR-146a-treated mSOD1 astrocytes increased the expression of this miRNA on naïve N9 microglial cells (Figure 8A). However, while MNs are not able to collect entire sEVs, microglia easily engulf them, as we showed (Pinto et al., 2017). Therefore, we isolated sEVs from the secretome of mSOD1 astrocytes untreated or treated with either pre-miR-146a or VS, and we investigated the presence of labeled sEVs in the N9 microglial cells 24 h after incubation. As shown in Figures 9A,B no differences in the number of sEVs incorporated by microglia were obtained when isolated from mSOD1 astrocyte secretome relatively to the WT astrocyte one. However, sEVs were found more densely internalized by microglia (*p* < 0.01) in the case of the pre-miR-146a strategy, than by VS. This feature may have accounted for the miR-146a elevation in the cells (*p* < 0.001, Figure 9C), due to sEV enriched cargo in miR-146a (Figure 5F).

Interestingly, both pre-miR-146a and VS treatment in mutated astrocytes produced sEVs able to prevent iNOS (*p* < 0.01, Figure 9D) and TNF-α (at least *p* < 0.001, Figure 9E) upregulation relatively to sEVs from untreated mSOD1 astrocytes. This is a very interesting result, if we consider that VS-derived secretome was unable to prevent microglia activation, while its isolated sEVs showed such property.

Data highlight the differential roles of the secretome (ineffective) and of the isolated sEVs (effective) from the VS-treated astrocytes in recovering microglia steady-state, while indicate to be independent of miR-146a content. However, in the case of astrocytes treated with pre-miR-146a, both the secretome and their isolated sEVs, suppressed microglia activation by a mechanism that is suggestive to depend on the paracrine influence of miR-146a expression.

## Discussion

Currently, there are no effective therapies for ALS and the recent advances in this field converges in the modulation of the inflammatory and toxic environment by controlling glial activation (Liu and Wang, 2017). Indeed, increased evidences have shown that reactive astrocytes impair the survival of MNs (Díaz-Amarilla et al., 2011; Meyer et al., 2014; Qian et al., 2017; Gomes et al., 2019).

We have recently identified the presence of a deregulated set of reactive markers in mSOD1 cortical astrocytes isolated from 7-day-old mSOD1 mice cultured for 13 DIV, which were similarly present in the symptomatic stage of the same animals (Gomes et al., 2019). The ALS-disease characteristic phenotype included a low GFAP expression, but increased levels of S100B, Cx43, vimentin and HMGB1. By showing the same markers identified in the symptomatic stage of the mSOD1 mice their characteristic fingerprint with low GFAP levels seems to be disease specific. This profile is in agreement with the previous one found in the SC of adult symptomatic mSOD1 rats (Díaz-Amarilla et al., 2011). Astrocyte reactivity is usually associated to elevated levels of GFAP (Eng and Ghirnikar, 1994). Indeed, GFAP was found elevated in the cerebrospinal fluid and in the SC of patients with ALS (Fujita et al., 1998; Benninger et al., 2016), as well as in isolated adult astrocytes using supplementation with growth factors from the mSOD1 mice (Tripathi et al., 2017). However, our data and that of others (Rossi et al., 2008; Díaz-Amarilla et al., 2011; Cunha et al., 2018; Gomes et al., 2020) are consistent with a downregulation of GFAP in the presence of reactive markers that also include increased Ki-67, together contributing to denominate astrocytes in ALS as having an aberrant phenotype. In a recent consensus statement, it is referred that the increase in GFAP expression do not correlate with increased injury, reactive response to pathological stimuli or altered functions of reactive astrocytes (Escartin et al., 2021). In conformity, it should be used instead a combination of molecular markers to qualify astrocytes as reactive and as having a disease-specific signature. In this context, cortical homogenates from the symptomatic mSOD1 mice and isolated astrocytes from such animal pups showed increased reactive astrocyte markers, decreased GLT-1 and GFAP levels, and the pioneering downregulated expression of miR-146a (Gomes et al., 2019, 2020). Such reduction was even present in the presymptomatic stage and could not be found in astrocytes from the spinal cord (Cunha et al., 2018), suggesting regional astrocyte heterogeneity and their different contribution to bulbar and spinal forms of ALS disease. Such findings indicate that different therapeutic approaches may be required, depending on the local of disease onset, and that replenishment of miR-146a in cortical astrocytes may reveal a promising strategy.

Interestingly, miR-146a-enriched stem cell secretome was recently suggested to have proangiogenic and anti-inflammatory properties (Waters et al., 2019). For that reason and given the importance of miR-146a in controlling astrocyte inflammation (Iyer et al., 2012), we decided to test the efficacy of pre-miR-146a and immunomodulatory compounds, such as GUDCA and VS (Falcão et al., 2017; Vaz et al., 2019) in reverting astrocyte reactivity and miR-146a replenishment. Our results demonstrated that VS induced upregulation of miR-146a and downregulation of its targets, while GUDCA did not change such levels. This is in line with our previous studies that demonstrated the ability of VS in modulating miR-146a expression (Falcão et al., 2017; Vaz et al., 2019). Furthermore, GUDCA reduced the expression of vimentin and Cx43, exerting a modulatory role on astrocyte reactivity, in accordance with our previous data (Fernandes et al., 2007). However, VS showed more broader effects, not only by restoring the levels of GFAP and decreasing vimentin and Cx43 expression, but also by downregulating S100B and HMGB1 levels, corroborating its anti-inflammatory properties previously observed in microglial models of ALS (Vaz et al., 2019) and Alzheimer’s disease (Falcão et al., 2017). To note that neither GUDCA nor VS showed to compromise cell viability. Then, we hypothesized if the attenuation of such aberrancy by these compounds might be related with the modulation of miR-146a expression.

To verify such supposition, we restored the normal levels of miR-146a by transfecting mSOD1 astrocytes with pre-miR-146a. Re-establishment of GFAP, vimentin, Cx43, HMGB1 and S100B toward control WT levels revealed its therapeutic potential. Actually, vimentin is not only an intermediate filament protein involved in cellular processes, such as cell adhesion, migration and proliferation, promoting wound healing (Ivaska et al., 2007), but it is also a known marker of immature astrocytes, associated with a reactive gliosis and leading to thicker bundles in the astrocytic processes (Cheng and Eriksson, 2017). Usually, the expression of GFAP replaces vimentin in differentiated astrocytes (Roybon et al., 2013). In the case of the cortical ALS astrocytes data suggest that they sustain an immature-like reactive phenotype. The correction of the low levels of miR-146a seems to contribute to abolish such ALS-disease phenotype of cortical astrocytes. Regulation of vimentin and GFAP levels by pre-miR-146a are in accordance with a study wherein miR-146a overexpression increased GFAP expression and attenuated proliferation, migration and tumorigenic potential of glioma cells (Mei et al., 2011; Xiao et al., 2015). We also found decreased S100B and HMGB1 expression gene levels in pre-miR-146a-treated mSOD1 astrocytes. This is in line with a previous study that suggested the increased astrocytic S100B expression as an early occurring event in ALS and a contributor for neurodegeneration (Serrano et al., 2017). These Authors observed that its inhibition in mSOD1 astrocytes decreased the expression of proinflammatory/reactive genes. Besides, this attenuation of astrocyte-inflammatory potential supports the role of miR-146a as a negative regulator of the NF-κB inflammatory pathway in astrocytes (Taganov et al., 2006; Iyer et al., 2012). Concerning Cx43, it is known to modulate proliferation, migration and differentiation of astrocytes (Homkajorn et al., 2010). Its upregulation was detected in mSOD1 astrocytes, corroborating previous studies in rodent models (Díaz-Amarilla et al., 2011; Cui et al., 2014), and the restoration of Cx43 normal levels in pre-miR-146a-treated mSOD1 astrocytes may associate to its ability to regulate inflammatory response and attenuate gliosis (Mei et al., 2011). Like GUDCA and VS, pre-miR-146a transfection also did not activate early/late apoptosis.

Proteomic analysis showed that pre-miR-146a induced an upregulation of the NCF1 protein that we hypothesize to be an attempt to control the reactive/immune response in mSOD1 astrocytes, as demonstrated in chronic inflammatory disorders (Holmdahl et al., 2016; Zhao et al., 2017). ZBTB10 was found elevated during gliogenesis and linked to ZBTB20 for differentiation and maturation of neocortical astrocytes (Nagao et al., 2016; Araújo, 2019). Moreover, ZBTB10 was also suggested to be associated to DNA damage repair at telomeres (Bluhm et al., 2019). As for HMGN2, it belongs to a family of proteins associated to astrocyte differentiation during development (Nagao et al., 2014). Both ZBTB20 and HMGN2 may be relevant protein targets to be used toward the recovery of the neuroprotective phenotype by the ALS cortical astrocytes which, in our view, deserve to be explored. The observed upregulation of ALS2CL, by regulating the alsin-mediated endosome, might induces sEV formation (Hessvik and Llorente, 2018) and the activation of the autophagy-endolysosomal system (Otomo et al., 2012). Together with the PZP upregulation associated to a sustained extracellular proteostasis and inhibition of misfolded protein accumulation (Cater et al., 2019), they might act together to promote mSOD1 clearance from astrocytes by sEVs and autophagy. Indeed, PZP elevation was found in senile plaques and in glial cells in Alzheimer’s disease, possibly having a protective role (Ijsselstijn et al., 2011; Nijholt et al., 2015).

To assess whether reversal of mSOD1 astrocyte aberrancies by VS was mediated via miR-146a regulation, we depleted its expression in WT astrocytes with a miR-146a inhibitor, before using VS. Anti-miR-146a-transfected WT astrocytes acquired the aberrant phenotype that characterize ALS cortical astrocytes. Interestingly, VS was not only able to restore miR-146a levels in such condition, but also to revert GFAP, S100B and vimentin toward steady-state levels. In sum, our results indicate that targeting downregulated miR-146a, either with pre-miR-146a or VS, constitutes a good strategy to counteract the atypical reactivity of ALS cortical astrocytes associated to the miR-146a depletion.

Given the established contribution of ALS astrocytes on MN death and the possible involvement of astrocytic Ca^2+^ dysregulation in this process (Kawamata et al., 2014), we investigated its presence and if it was modulated by either pre-miR-146a or VS treatments. Our results revealed a baseline increase of intracellular Ca^2+^ in mSOD1 astrocytes that was prevented by VS co-incubation, together with elevated frequency of Ca^2+^ transients after glutamate stimulation, what may impact on synapse integrity (Guerra-Gomes et al., 2017). Astrocytes possess glutamate-sensitive ion channels and can respond to glutamate with a fast and oscillatory elevation of cytoplasmic free Ca^2+^ by autocrine and paracrine effects (Cornell-Bell et al., 1990). We hypothesize that the excess of Ca^2+^ in mSOD1 astrocytes may derive from ER stores, as observed in the SC of mSOD1 mice (Kawamata et al., 2014), or be caused by the aberrant Cx43 overexpression (Almad et al., 2016), inducing alterations in neuronal excitability, synaptic transmission and plasticity (Lu et al., 2010). As so, pre-miR-146a was able to reduce the number of [Ca^+^]i transients in mSOD1 astrocytes and to downregulate Cx43 expression. Previous studies observed the reduction in Ca^2+^ responses in the presence of a Cx43 blocker in ALS astrocytes (Almad et al., 2016, 2020).

Astrocytes are highly secretory cells, with their secretome containing hundreds of molecules. In ALS, it is accepted that astrocytes have a paracrine contribution to neurodegeneration and microglial activation, but the mechanisms associated are poorly explored (Pehar et al., 2017). As a component of secretome, sEVs mediate the interaction between neurons and glial cells and are responsible for the delivery of proteins, lipids, mRNAs and miRNAs in the recipient cell, being considered potential vehicles for drug delivery (Basso and Bonetto, 2016). Our observation that mSOD1 astrocytes release sEVs with low miR-146a content is in line with our previous findings (Gomes et al., 2020). In the same study, we established that mSOD1 astrocytes contribute to the neuronal degeneration when these cells are co-cultured with ALS MNs. Indeed, mSOD1 astrocytes selectively provoke toxicity to MNs (Di Giorgio et al., 2007; Nagai et al., 2007; Díaz-Amarilla et al., 2011; Haidet-Phillips et al., 2011; Gomes et al., 2019) and their secretome was found to play an important role (Nagai et al., 2007).

We observed that early and late apoptosis were induced by the secretome from the pathological ALS astrocytes, leading to a reduced expression of synaptophysin, PSD-95, dynein, and kinesin in MNs, thus compromising synaptic and axonal dynamics. Synaptophysin, a presynaptic membrane protein, was found reduced in MNs from mSOD1 mice and shown to contribute to motor deficits (Zang et al., 2005). The postsynaptic PSD-95, also important for synaptic plasticity (Zhang and Lisman, 2012), has been found reduced in neurodegenerative diseases, including ALS (Fogarty, 2019). Contribution of ALS astrocytes to synaptic failure is an established feature (Casas et al., 2016). In particular, these cortical mSOD1 astrocytes showed to reduce neurite length and synaptophysin expression in coculture with NSC-34 hSOD1 WT MN-like cells (Gomes et al., 2019). Defects in the anterograde and retrograde transports by kinesin and dynein, respectively, also occur in ALS neurodegeneration in patients and in mSOD1 mice (De Vos and Hafezparast, 2017). Inefficient anterograde transport was shown to reduce the transport of synaptic vesicle precursors, contributing to the decrease in synaptic vesicle density (Yonekawa et al., 1998) that is reflected by the reduced expression of the synaptic proteins, as we observed. Secretome from pre-miR-146a-treated mSOD1 astrocytes, but not from VS-treated ones, restored PSD-95 and kinesin levels found deregulated by the pathological paracrine signaling over WT MNs.

We identified for the first time that FGFR1 and FGFR3 gene expression levels, receptors of fibroblast growth factor-2 (FGF2), increased in MNs upon the secretome from mSOD1 astrocytes. FGF2 is highly produced in reactive astrocytes after injury and ischemia (Reilly and Kumari, 1996; Clarke et al., 2001) and their elevation was noticed in the CSF from ALS patients (Johansson et al., 2003; Ekestern, 2004). The upregulation of FGFR1 and FGFR3 identified in our study by the secretome from mSOD1 astrocytes may contribute to axonal damage involving the ERK/MAPK signaling (Huang et al., 2020). Instead, the secretome derived from pre-miR-146a-treated mSOD1 astrocyte was unique in decreasing the expression of these receptors and together with VS prevented the induced MN apoptosis upon treatment with the pathological astrocyte-derived secretome.

Motor neuron proteomic analysis revealed that pre-miR-146a increases the E2 component of the pyruvate dehydrogenase complex encoded by the *Dlat* gene (Goguet-Rubio et al., 2016), leading to the production of ATP that is required to continuously provide energy to maintain normal MN function with a high relevance in ALS (Vandoorne et al., 2018). By downregulating ATP1F1, pre-miR-146a may also ameliorate mitochondrial respiratory chain dysfunction because it inhibits ATPase activity (Chen et al., 2014), contributing to sustain ALS MN mitochondrial functionality (Li et al., 2010; Chen et al., 2014), and thus preventing the apoptosis induced by the secretome from pre-miR-146a-treated mSOD1 astrocytes. In conformity, the decreased expression of FTSJ1, as we found, was also associated to the inhibition of apoptosis in cancer (He et al., 2020). It was interesting to observe a reduction of SLCA13, since this transporter was identified as a risk factor for frontotemporal dementia (Sirkis et al., 2019). By being reduced, it prevents APP processing (Teranishi et al., 2015) and the interaction between SOD1 and APP, which are known to lead to synaptic dysfunctions and associated neuroinflammation and neurodegeneration events in ALS (Rabinovich-Toidman et al., 2015). We also found downregulation of RBPMS in MNs following the interaction with the secretome from pre-miR-146a-treated mSOD1 astrocytes. Its transcript was found downregulated in the axonal transcriptome from MNs overexpressing human mSOD1 (Nijssen et al., 2018) and showed to inversely affect synapse density (Hornberg et al., 2013).

VS treatment exceeded the beneficial effects of pre-miR-146a treatment by promoting MN functionality through upregulation of synaptophysin, PSD-95, dynein and kinesin transcripts and by avoiding early cell death by apoptosis. This is in line with a previous study describing vinyl sulfones as neuroprotective agents in the treatment of Parkinson’s disease (Woo et al., 2014; Choi et al., 2019). Increased levels of miR-146a in MNs were observed after treatment with the secretome from the pre-miR-146a-treated mSOD1 astrocytes, but not with that from VS treatment. We may hypothesize that the enriched content of the secretome or their isolated sEVs in miR-146a may have accounted for such MN upregulation and eventually the observed benefits. Indeed, previous studies demonstrated that miR-146a is important for neuronal survival and differentiation (Qu et al., 2019; Fregeac et al., 2020), as well as for axonal growth (Jia et al., 2016). This is not the case of VS, suggesting that its paracrine neuroprotective effect is not exclusively dependent on sEV-derived miR-146a. In contrast, their effectiveness might be related with soluble miR-146a or other neuroprotective factors, an interesting hypothesis to be explored in future studies.

The secretome from mSOD1 astrocytes also switched microglia to a pro-inflammatory subtype by increasing iNOS and TNF-α mRNA expression, in accordance with studies where mSOD1 astrocytes led to the activation of microglia, thus contributing to disease progression (Yamanaka et al., 2008). Moreover, we observed the activation of early/late apoptosis and an increase of FGFR3 expression. Injury was shown to upregulate the FGFR3 expression in both astrocytes and microglia and FGF2 appears to have a dual role, by firstly inducing gliosis and secondly neuroprotection (Goddard et al., 2002). Here, we may speculate that, as observed for damaged neurons, cortical ALS astrocytes release FGF2 and enhances the expression of FGFR3, which has been associated to neuroprotection against glutamate toxicity and increased microglial migration and phagocytosis (Noda et al., 2014). Nevertheless, excessive neuronal phagoptosis also contributes to a range of neurodegenerative diseases, including ALS (Brown and Neher, 2014). Therefore, if we consider that the secretome derived from both pre-miR-146a and VS-treated astrocytes reduces FGFR3 levels in microglia, it may then prevent reactivity and excessive phagoptosis. However, only the secretome derived from pre-miR-146a -treated astrocytes averted microglial inflammatory activation.

To better understand the effects produced by the secretome from mSOD1 astrocytes treated with pre-miR-146a on microglia we performed proteomic analysis. Among the effects produced we noticed a reduction in *Cdk1* that may have contributed to the decrease of iNOS and TNF-α transcripts by the modulated secretome. Such proinflammatory signaling activation, characteristic of a polarized activated microglia (Sheng et al., 2011), was shown to be prevented by CDK inhibitors (Leitch et al., 2009; Skovira et al., 2016). Similarly contributing to counteract the activation of microglia is the reduction we found on *Adamts1*, once its upregulation was identified in LPS-activated microglia (Chiu et al., 2013) and associated to a tumor inflammatory response (Rodriguez-Baena et al., 2018). These findings indicate that pre-miR-146a contributes to a less polarized microglia phenotype. Regarding *Igdcc4*, also described as being involved in immune response (Sanz-Pamplona et al., 2014) and a biomarker in hepatocellular carcinoma (Zweerink et al., 2020), further studies should investigate its precise role in microglia.

Now going for the interaction of sEVs on microglia, though we did not observe changes in the concentration and size of sEVs derived from pre-miR-146a-treated astrocytes, an increased internalization of sEVs in microglia was noticed, when compared with sEVs derived from the pathological untreated mSOD1 astrocytes or VS-treated ones. This increased uptake by the microglial cells is not surprising since it was demonstrated that miR-146a leads to the upregulation of several microglia surface receptors (Saba et al., 2012) that most likely enable the uptake of sEVs. Also, previous studies demonstrated that microglia are one of the neural cells that show higher ability for sEV incorporation (Fitzner et al., 2011; Pinto et al., 2017; Xia et al., 2019).

In a previous study we evidenced that sEVs derived from the secretome of mSOD1 MNs with an enriched cargo in miR-124 were able to activate microglia (Pinto et al., 2017). Similarly, our sEVs depleted in miR-146a and isolated from the secretome of mSOD1 cortical astrocytes led to increased gene expression of iNOS and TNF-α. We further demonstrated that such effect was counteracted when we used pre-miR-146a or VS-treatment in mSOD1 cortical astrocytes. This is in line with studies evidencing that EVs containing miR-146a attenuate inflammation, supporting tissue repair and regeneration (Alexander et al., 2015; Waters et al., 2019; Wu et al., 2019). Moreover, miR-146a packaged into EVs and transferred into macrophages showed therapeutic efficacy against sepsis (Song et al., 2017), thus reinforcing its protective effects. Interestingly, despite not having increased cargo in miR-146a, sEVs isolated from the secretome of VS-treated mSOD1 astrocytes also demonstrated ability to switch the activated microglia into the steady-state phenotype, indicating that miR-146a is not the sole neuroprotective factor involved in the reestablishment of the healthy microglia state.

**FIGURE 10:**
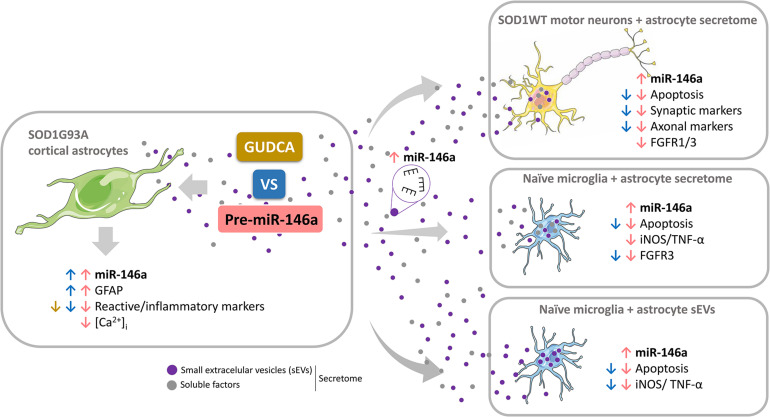
Schematic representation of the efficacy of miR-146a replenishing methods to recover the neuroprotective phenotype of aberrant mSOD1 cortical astrocytes, and in preventing toxic paracrine signaling toward motor neurons and microglia by the mediators released from the pathological astrocytes. Astrocytes were isolated from the cortex of SOD1-G93A (mSOD1) mice pups at 7-day-old and cultured for 13 days *in vitro*, showing an aberrant/reactive phenotype. Treatment with glycoursodeoxycholic acid (GUDCA) and dipeptidyl vinyl sulfone (VS) abrogated reactive markers, with additional re-establishment of glial fibrillary acidic protein (GFAP) and miR-146a by VS, evidencing their reparative ability. Transfection of mSOD1 astrocytes with pre-miR-146a also attenuated their phenotypic aberrancies and intracellular Ca^2+^ ([Ca^2+^]_i_) overload. Moreover, such treatment increased miR146a content in the cell-derived small extracellular vesicles (sEVs), and mediated miR-146a enrichment in SOD1-WT motor neurons (MNs) and naïve N9 microglial cell. Secretome from mSOD1 astrocytes increased early/late apoptosis and fibroblast growth factor receptor (FGFR) gene levels in MNs and microglia, effects that were prevented by pre-miR-146a or VS modulation. These strategies led to a secretome with preventable properties over the deregulation of synaptic dynamics and axonal transport upon the pathological extracellular milieu from mSOD1 astrocytes. The pre-miR-146a-treated cells also prevented microglia activation through their secretome or isolated sEVs, but in the case of VS only the isolated sEVs showed such property. Data reveal that both pre-miR-146a and VS-mediated miR-146a replenishment in mSOD1 cortical astrocytes are promising approaches to recover the neuroprotective phenotype of ALS cortical astrocytes and the microglia and MN homeostatic balance in the disease. [Ca^2+^]i, intracellular calcium; GFAP, glial fibrillary acidic protein; FGFR, fibroblast growth factor receptor; GUDCA, glycoursodeoxycholic acid; iNOS, inducible nitric oxide synthase; miR-146a, miRNA-146a; SOD1, superoxide dismutase 1; TNF-α, tumor necrosis alpha; VS, dipeptidyl vinyl sulfone.

In sum, our study highlights that the downregulation of miR-146a in mSOD1 cortical astrocytes is responsible, at least in part, by the reactivity and aberrant features of these glial cells. We propose pre-miR-146a transfection and VS treatment as efficient strategies to induce miR-146a upregulation in cortical ALS astrocytes toward cell revival through the abrogation of their reactive/aberrant phenotype. These potential therapeutic approaches have additional advantages in re-establishing microglia and MN homeostatic states by paracrine signaling. Comparative effects of the miR-146a replenishing methods, i.e., pre-miR-146a, GUDCA and VS, in the ability to recovery of the neuroprotective phenotype of cortical astrocytes from mSOD1 mice, and the resulting paracrine signaling on WT MNs and N9 microglial cells are schematically represented in Figure 10. Overall, we consider sEV-enrichment in VS and pre-miR-146a cargoes as opportunities for neuro-glial restorative therapy in ALS disease.

## Data Availability Statement

The datasets presented in this study are available in online repositories. The names of the repository/repositories and accession number(s) can be found in the article/ Supplementary Material.

## Ethics Statement

The animal study was reviewed and approved by Portuguese National Authority (General Direction of Veterinary) (Directives 86/609/EU and 2010/63/EU, Recommendation 2007/526/CE, European Convention for the Protection of Vertebrate Animals used for Experimental or Other Scientific Purposes ETS 123/Appendix A) and Portuguese Laws on Animal Care (Decreto-Lei 129/92, Portaria 1005/92, Portaria 466/95, Decreto-Lei 197/96, and Portaria 1131/97), Portugal; Ethics Committee of the Instituto de Medicina Molecular João Lobo Antunes (IMM), Faculdade de Medicina, Universidade de Lisboa, Portugal.

## Author Contributions

DB conceived the project. MB, AV, and DB planned and designed the experiments. MB performed most of the experimental work, analyzed data, and drafted the manuscript. AV, CG, and CS participated in the preparation and maintenance of primary astrocyte cultures and small extracellular vesicles characterization. SV planned and analyzed data of calcium experiments. CP and JG-R performed experimental work and analyzed data of calcium experiments. RM and LC synthesized the VS compound. MB, AV, and DB interpreted results and wrote the final version of the manuscript. DB supervised all aspects and edited the manuscript. All authors contributed to the article and approved the submitted version.

## Conflict of Interest

The authors declare that the research was conducted in the absence of any commercial or financial relationships that could be construed as a potential conflict of interest.

## Footnotes

1 http://www.ebi.ac.uk/pride/archive/projects/PXD024294
