# Supplementary material for: Recovery of Depleted miR-146a in ALS Cortical Astrocytes Reverts Cell Aberrancies and Prevents Paracrine Pathogenicity on Microglia and Motor Neurons
Source: Front Cell Dev Biol. 2021 Apr 23;9:634355. doi: 10.3389/fcell.2021.634355 (PMC8103001; doi:10.3389/fcell.2021.634355)
Supplement: Supplementary file 9 [file Data_Sheet_1.PDF]

Supplementary Tables

Supplementary Table S1 | Gene and miRNA expression of WT astrocytes only treated with X-tremeGENE™ HP DNA Transfection Reagent (mock control) relatively to WT astrocytes modulated with Anti-miR™ Negative Control, and of mSOD1 astrocytes only treated with X-tremeGENE™ HP DNA Transfection Reagent relatively to mSOD1 astrocytes modulated with Pre-miR™ Negative Control.

Markers	WT (mock)	WT + negative control anti-miRNA	mSOD1 (mock)	mSOD1 + negative control pre-miRNA
**mRNA**				
IRAK1	1.00	0.94 ± 0.10	1.00	1.36 ± 0.59
TRAF6	1.00	1.13 ± 0.09	1.00	1.10 ± 0.15
S100B	1.00	1.09 ± 0.05	1.00	0.92 ± 0.21
Vimentin	1.00	1.12 ± 0.02	1.00	0.92 ± 0.09
Cx43	1.00	0.95 ± 0.03	1.00	0.86 ± 0.05
**microRNA**				
miR-146a	1.00	0.97 ± 0.07	1.00	1.11 ± 0.18

Results are expressed as fold change (MEAN ± SEM) from at least three independent experiences. Paired two-tailed Student's *t*-test was used to compare the means between mock control and respective modulated astrocytes. No significant differences were found. WT, wild type; mSOD1, SOD1G93A mutation; IRAK1, interleukin-1 receptor-associated kinase 1; TRAF6, TNF receptor associated factor 6; S100B, S100 calcium binding protein B; Cx43, connexin 43; miR-146a, miRNA-146a.

Supplementary Table S2 | List of antibodies used for immunocytochemistry (ICC) or western blot (WB).

Antibodies	Source	Species	Dilution	
			ICC	***WB***
Anti-GFAP	Sigma-Aldrich, G9269	Rabbit	1:500	1:500
Anti-S100B	AbCam, ab52642	Rabbit		1:500
Anti-Cx43	Santa Cruz Biotechnology, sc13558	Mouse		1:200
Anti-Alix A39	Cell Signaling, #2171	Mouse	---	1:500
Anti-Flotillin-1	BD Biosciences, 6108	Mouse	---	1:500
Anti-β-actin	Sigma, A5441	Mouse	---	1:5000
Anti-rabbit HRP	Santa Cruz Biotechnology, sc2004	---	---	1:5000
Anti-mouse HRP	Santa Cruz Biotechnology, sc2005	---	---	1:5000
Anti-goat HRP	Santa Cruz Biotechnology, sc2768	---	---	1:4000
Anti-rabbit AlexaFluor 594	Invitrogen Corporation, A-11005	---	1:1000	---

Cx43, connexin 43; GFAP, glial fibrillary acidic protein; S100B, S100 calcium-binding protein B; HRP, horseradish peroxidase.

Supplementary Table S3 | List of primer sequences used in RT-qPCR.

Gene	Forward primer sequence	Reverse primer sequence
Irak1	5’ GAGACCCTTGCTGGTCAGAG 3’	5’ GCTACACCCACCCACAGAGT 3’
Traf6	5’AAAGCGAGAGATTCTTTCCCTG 3’	5’ ACTGGGGACAATTCACTAGAGC 3’
Hmgb1	5’ CTCAGAGAGGTGGAAGACCATGT 3’	5’ GGGATGTAGGTTTTCATTTCTCTTTC 3’
Tnf-α	5’ TACTGAACTTCGGGGTGATTGGTCC 3’	5’ CAGCCTTGTCCCTTGAAGAGAACC 3’
S100b	5’ GAGAGAGGGTGACAAGCACAA 3’	5’ GGCCATAAACTCCTGGAAGTC 3’
Vimentin	5’ CGGAAAGTGGAATCCTTGCA 3’	5’ CACATCGATCTGGACATGCTGT 3’
Cx43	5’ ACAGCGGTTGAGTCAGCTTG 3’	5’ GAGAGATGGGGAAGGACTTGT 3’
iNos	5’ ACCCACATCTGGCAGAATGAG 3’	5’ AGCCATGACCTTTCGCATTAG 3’
Synaptophysin	5’-GACGTTGGTAGTGCCTGTGA-3’	5’-GCACAGGAAAGTAGGGGGTC-3’
Dlg4	5’-GAGGCTGGCGGCCAGTACACCAG-3’	5’-ACAGAGCAGGCGGTCAG-3’
Dynein	5’-GCCTCAGTCTCTGTCCCATC-3’	5’-AAGTCCTGGGGTAAGGTGCT-3’
Kif5b	5’-GGTCCTACAGTTGCCACCTA-3’	5’-ATTGAAATACGCCAGGCCCA-3’
Fgfr1	5’-CAGATGCACTCCCATCCTCG-3’	5’-TCTCTCCTCCCACTTTAGCCA-3’
Fgfr3	5’-GTGGTAGGTCAGGTGGTTGG-3’	5’-GATCACCCGGTCTTCCACTC-3’
β-actin	5’ GCTCCGGCATGTGCAA 3’	5’ AGGATCTTCATGAGGTAGT 3’

Irak1, interleukin-1 receptor-associated kinase 1; *Traf6*, TNF receptor associated factor 6, *Hmgb1*, high-mobility-group-box-1 protein; *Tnf-α*, tumor necrosis factor alpha; *S100b*, S100 calcium binding protein B; *Cx43*, connexin 43; *iNos*, inducible nitric oxide synthase; *Fgfr*, fibroblast growth factor receptor; *Dlg4* encodes for the protein postsynaptic density-95; *Kif5b* encodes for the protein kinesin.

Supplementary Table S4 | List of proteins differentially expressed and identified as hits by proteomic analysis of the pre-miR-146a-treated mSOD1 astrocytes relatively to the untreated cells.

Gene symbol	Protein ID	Description	AveExp	Log_2 (FC)
Als2cl	Q60I26	ALS2 C-terminal-like protein	23.63	1.25
Chd8	F7AL76|Q09XV5	Chromodomain-helicase-DNA-binding protein 8	25.28	1.26
Ezh1	A0A0R4J1C0|P70351	Histone-lysine N-methyltransferase	23.91	1.09
Gtpbp6	A0A0R4J0Z0|Q3U6U5	Putative GTP-binding protein 6	20.79	1.13
Hmgn2	A3KGL9|F6W687|P09602 Q5XK38	Non-histone chromosomal protein HMG-17	26.15	-1.04
Lrch4	H3BLL3|Q921G6	Leucine-rich repeat and calponin homology domain-containing protein 4	24.93	1.31
Ncf1	F8WH69|Q09014|S4R293	Neutrophil cytosol factor 1	26.36	1.32
Pzp	Q61838	Pregnancy zone protein	24.42	1.23
Thg1l	Q9CQT0|Q9CY52	tRNA(His) guanylyltransferase	25.38	1.12
Zbtb10	E9Q8X5	Zinc finger and BTB domain-containing 10	22.38	2.04

Gene Symbol, Protein identification (ID) and Description are according with the UniprotKB database and the computationally mapped potential isoforms are indicated in Protein ID column. Average expression (AveExp, signal_sum) and log_2 (fold change, FC) values are indicated. Functional classification of the gene lists for Mus musculus.

Supplementary Table S5 | | List of proteins differentially expressed and identified as hits by proteomic analysis of WT motor neurons incubated with the secretome from pre-miR-146a-treated mSOD1 astrocytes relatively to the untreated cells.

Gene symbol	Protein ID	Description	AveExp	Log_2 (FC)
Atpif1	E9PV44|O35143	ATP synthase F1 subunit epsilon	27.10	-1.05
Dlat	Q8BMF4	Dihydrolipoyllysine-residue acetyltransferase component of pyruvate dehydrogenase complex, mitochondrial	28.40	1.02
Ftsj1	Q8CBC7|Q8JZY1	Putative tRNA (cytidine(32)/guanosine(34)-2'-O)-methyltransferase	21.70	-1.20
Rbpms	Q9WVB0|Q9WVB0-2 T1ECW4	RNA-binding protein with multiple splicing	22.27	-1.32
Slc2a13	Q3UHK1	Proton myo-inositol cotransporter	21.06	-1.47

Gene Symbol, Protein identification (ID) and Description are according with the UniprotKB database and the computationally mapped potential isoforms are indicated in Protein ID column. Average expression (AveExp, signal_sum) and log_2 (fold change, FC) values are indicated. Functional classification of the gene lists for Mus musculus.

Supplementary Table S6 | List of proteins differentially expressed and identified as hits by proteomic analysis of naïve microglia incubated with the secretome from pre-miR-146a-treated mSOD1 astrocytes relatively to the untreated ones.

Gene symbol	Protein ID	Description	AveExp	Log_2 (FC)
Adamts1	E9PY08|P97857	A disintegrin and metalloproteinase with thrombospondin motifs 1	24.00	-1.02
Cdk1	P11440	Cyclin-dependent kinase 1	28.56	-1.04
Igdcc4	E9QAQ0	Immunoglobulin superfamily DCC subclass member 4	24.08	-1.04

Gene Symbol, Protein identification (ID) and Description are according with the UniprotKB database and the computationally mapped potential isoforms are indicated in Protein ID column. Average expression (AveExp, signal_sum) and log_2 (fold change, FC) values are indicated. Functional classification of the gene lists for Mus musculus.
