# Supplementary material for: Recovery of Depleted miR-146a in ALS Cortical Astrocytes Reverts Cell Aberrancies and Prevents Paracrine Pathogenicity on Microglia and Motor Neurons
Source: Front Cell Dev Biol. 2021 Apr 23;9:634355. doi: 10.3389/fcell.2021.634355 (PMC8103001; doi:10.3389/fcell.2021.634355)
Supplement: Supplementary file 10 [file Data_Sheet_2.PDF]

Supplementary Figures

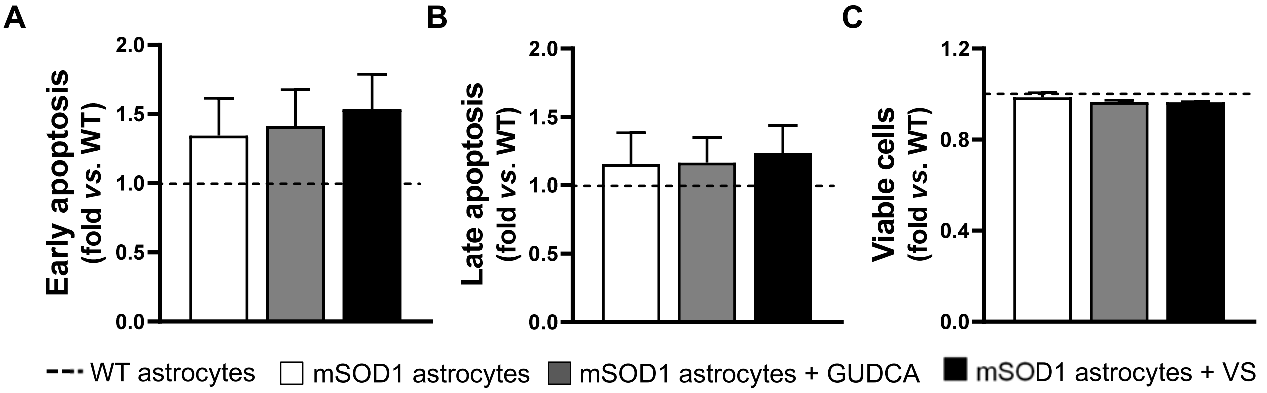

Supplementary Figure S1. Treatment of mSOD1 astrocytes with GUDCA and VS does not modify their viability. Astrocytes were isolated from the cortex of SOD1-G93A (mSOD1) and wild type (WT) mice pups at 7 day-old and cultured for 13 days in vitro. Treatment with glycoursodeoxycholic acid (GUDCA) or dipeptidyl vinyl sulfone (VS) was performed in mSOD1 astrocytes. **(A)** Early apoptotic cells (Annexin V-PE positive and 7-AAD negative), **(B)** late apoptotic/necrotic cells (Annexin V-PE and 7-AAD positive) and **(C)** viable cells (Annexin V-PE and 7-AAD negative) in WT MNs were assessed by Guava Nexin® Reagent. Results are mean (± SEM) fold change *vs.* WT astrocytes from at least three independent experiments. One-way ANOVA followed by Bonferroni post-hoc test was used.

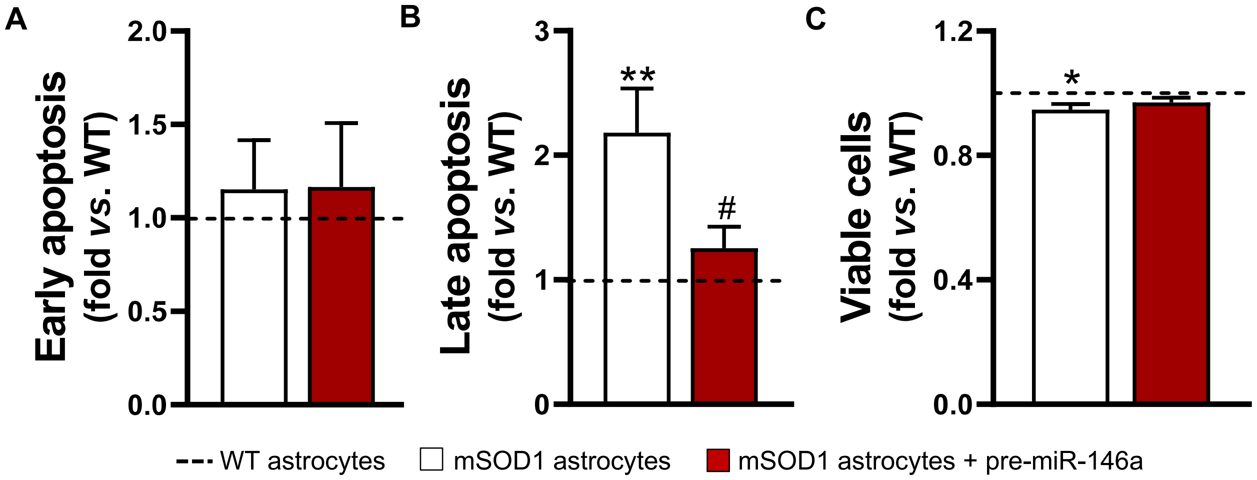

Supplementary Figure S2. Astrocytes from mSOD1 mice are susceptible to cell transfection showing increased late apoptosis and less viable cells than WT astrocytes, features that disappear after transfection with pre-miR-146a. Astrocytes were isolated from the cortex of SOD1-G93A (mSOD1) and wild type (WT) mice with 7 day-old and cultured for 13 days in vitro. Transfection with pre-miR-146a was performed in mSOD1 astrocytes. **(A)** Early apoptotic cells (Annexin V-PE positive and 7-AAD negative), **(B)** late apoptotic/necrotic cells (Annexin V-PE and 7-AAD positive) and **(C)** viable cells (Annexin V-PE and 7-AAD negative) in WT MNs were assessed by Guava Nexin® Reagent. Results are mean (± SEM) fold change *vs*. WT astrocytes from at least three independent experiments. $^*p < 0.05$ and $^{**}p < 0.01$ *vs*. WT astrocytes, $^{\#}p < 0.05$ *vs*. non-modulated mSOD1 astrocytes. One-way ANOVA followed by Bonferroni post-hoc test was used.

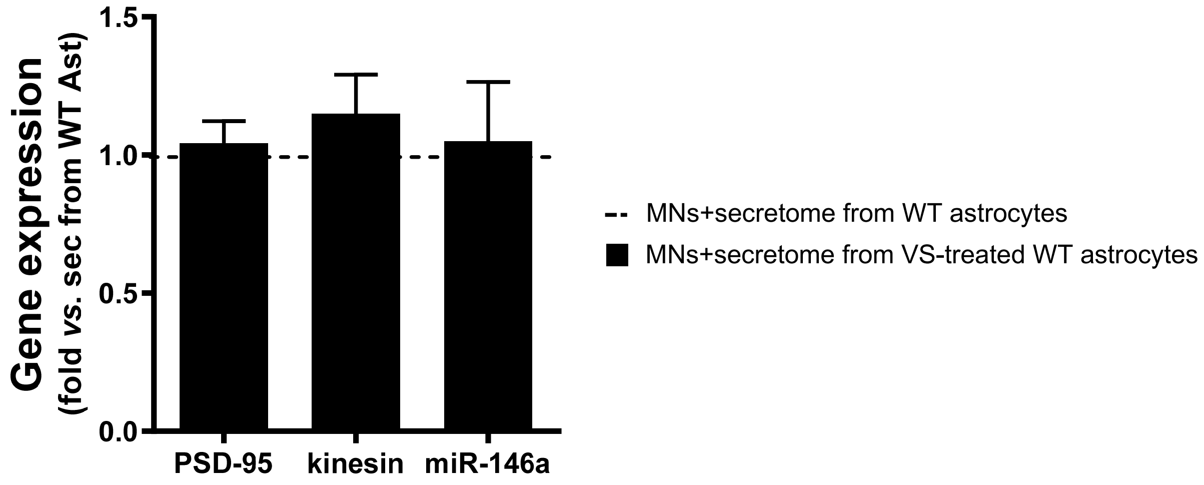

Supplementary Figure S3. Gene expression of axonal and synaptic proteins, as well as miR-146a levels in WT MNs are not modified by the secretome from VS-treated WT astrocytes. Astrocytes (Ast) were isolated from the cortex of 7-day-old wild type (WT) mice, cultured for 13 days in vitro, and treated with dipeptidyl vinyl sulfone (VS). Cell secretome (sec) was isolated and incubated in WT NSC-34 motor neuron (MN)-like cells for 48 h. Analysis of post-synaptic protein 95 (PSD-95), axonal transport kinesin protein and microRNA(miR)-146a in WT MNs were assessed by RT-qPCR. Expression of β-actin was used as an endogenous control for PSD-95 and kinesin analysis, and SNORD110 was used as reference gene for miR-146a analysis. Results are mean (± SEM) fold change *vs*. non-treated WT ACM from at least three independent experiments. Two-tailed Student's *t*-test was used.
